# Multiparametric imaging of spatio-temporal cAMP signaling, transmembrane potential, and intracellular calcium in the intact heart

**DOI:** 10.1016/j.isci.2026.114779

**Published:** 2026-01-22

**Authors:** I-Ju E. Lee, Jessica L. Caldwell, Lilian R. Mott, Jorge L. Gonzalez, Lianguo Wang, Crystal M. Ripplinger

**Affiliations:** 1 Department of Biomedical Engineering, University of California, Davis, Davis, CA, USA; 2 Department of Pharmacology, University of California, Davis, Davis, CA, USA

**Keywords:** molecular physiology, bioengineering, experimental models in systems biology

## Abstract

The spatiotemporal dynamics of intracellular second messengers and signaling molecules, including cyclic adenosine monophosphate (cAMP), have been studied extensively in isolated cardiomyocytes using Förster resonance energy transfer (FRET)-based reporters. However, approaches for linking cellular signaling to tissue-level function are limited. Here, we present a multiparametric imaging system that simultaneously records four wavelengths to visualize cAMP activity alongside real-time transmembrane potential and intracellular Ca^2+^ in intact hearts from a cardiac-specific cAMP reporter mouse. We showed that cAMP is strongly and heterogeneously activated throughout the heart (atria and ventricles) in response to pharmacological or nerve-activated β-adrenergic receptor stimulation, the time course of which matches heart rate, action potential, and Ca^2+^ responses. This multiparametric imaging system will provide insight into the relationship between cAMP signaling, electrophysiology, and arrhythmogenesis at high spatiotemporal resolution.

## Highlights

• Validated multiparametric imaging of cAMP, *V*_m_, and Ca^2+^ in intact hearts
• cAMP FRET acquired with and without excitation-contraction uncoupling
• β-AR stimulation triggers heterogeneous cAMP while tracking HR, APD, and Ca^2+^
• Quantification of sympathetic and parasympathetic responses in atria and ventricles

## Graphical abstract

**Figure:**
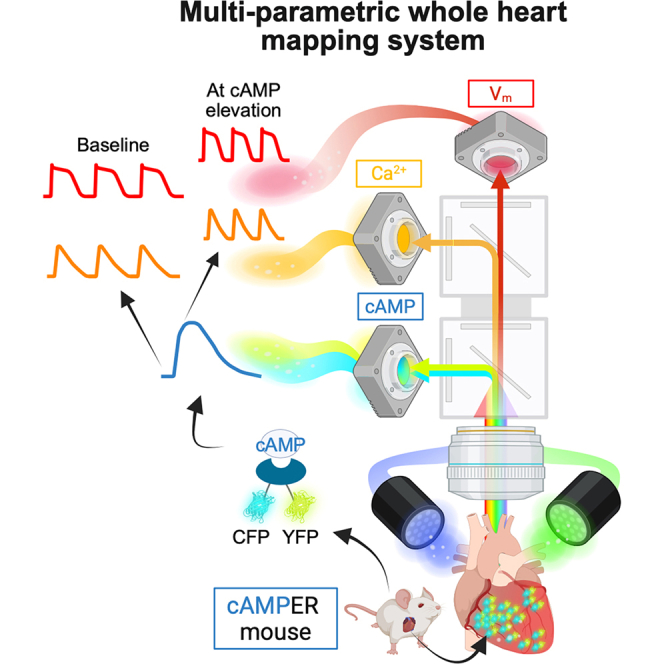

## Introduction

Genetically encoded Förster resonance energy transfer (FRET)-based biosensors have been used to understand the spatiotemporal dynamics of second messengers and signaling molecules in live cells. FRET enables continuous, non-invasive monitoring while preserving physiological context. A number of biosensors have been created to measure cyclic nucleotide generation or sub-cellular localization (e.g., cyclic adenosine monophosphate [cAMP] and cyclic guanosine monophosphate [cGMP]),^1^^,^^2^^,^^3^^,^^4^ kinase activity (e.g., protein kinase A [PKA] and Ca^2+^ calmodulin kinase II [CaMKII]),^5^^,^^6^^,^^7^ and associations between the sarcoplasmic reticulum ATPase (SERCA) pump and phospholamban^8^ in live cardiomyocytes. While FRET sensors offer valuable insights into sub-cellular compartmentalization, target activity, and nanodomain signaling, isolated cardiomyocytes have limitations when removed from the intact heart. For example, it is unclear if certain electrophysiological events, including early or delayed afterdepolarizations in an individual myocyte will overcome the source-sink mismatch to trigger premature ventricular complexes in tissue.^9^ Moreover, isolated cardiomyocytes are often pooled from throughout the heart (e.g., left ventricular [LV] and right ventricular [RV] myocytes are combined), and the expression of ion channels and other signaling proteins can change significantly if cells are cultured.^10^ These approaches also fail to account for intrinsic regional heterogeneity within the myocardium, as gradients in ion channel expression and sympathetic innervation, such as those between the ventricular base and apex, are lost when cells are dissociated and studied in isolation.^11^

Cardiac optical mapping is a fluorescence-based technique which offers unprecedented spatial resolution and real-time visualization of electrical activity across the surface of intact Langendorff-perfused hearts.^12^ This method allows for direct, contactless recording of transmembrane potential (*V*_m_) and optical action potentials, facilitating the measurement of electrical activity and the identification of tissue heterogeneities that can lead to arrhythmias.^13^ Additionally, optical mapping can directly image intracellular calcium (Ca^2+^_*i*_) transients, capturing the rise and fall of Ca^2+^_*i*_ that triggers the contraction and relaxation of cardiac muscle cells.^14^ Despite these advantages, cardiac optical mapping gives little insight into the underlying molecular signaling mechanisms. To gain cellular and molecular insight, recent advances in tissue clearing have allowed for labeling of proteins of interest in intact hearts,^15^^,^^16^ but relating real-time molecular signaling events to whole-heart function remains challenging.

To address this gap, we initially chose to image cAMP at the whole-heart level because it is a crucial second messenger in the adrenergic signaling cascade, and there is limited understanding of macroscale heterogeneity of cAMP signaling throughout the heart and its contribution to arrhythmogenesis. Here, we generated a cardiac-specific cAMP reporter mouse^17^ expressing a FRET-based Epac-mediated cAMP biosensor (termed *CAMPER* mouse) and developed an integrated whole-heart optical imaging system capable of simultaneous FRET imaging of cAMP activity along with dual optical mapping of *V*_m_ and Ca^2+^_*i*_. This approach uses cAMP as a readout of sympathetic signaling, and records *V*_m_ and Ca^2+^_*i*_ as concomitant functional responses. We also show real-time cAMP and *V*_m_ activation in the atria and locate the earliest activation point, allowing us to determine the cAMP response in sinoatrial nodal (SAN) tissues. To test the heart’s response to physiological nerve activity, we demonstrate the spatiotemporal kinetics of cAMP generation in response to chemically induced sympathetic nerve stimulation (SNS), which is known to have important functional differences compared to *in vitro* agonist application.^18^ We also demonstrate the spatiotemporal kinetics of cAMP reduction during parasympathetic activation. Overall, our multiparametric imaging system provides a powerful tool to study the spatial heterogeneity of cAMP activity and its electrophysiological responses, offering new insights into the mechanisms that may underlie arrhythmia susceptibility in the diseased heart.

## Results

### Quadruple-parametric imaging system set up

**Figure 1:**
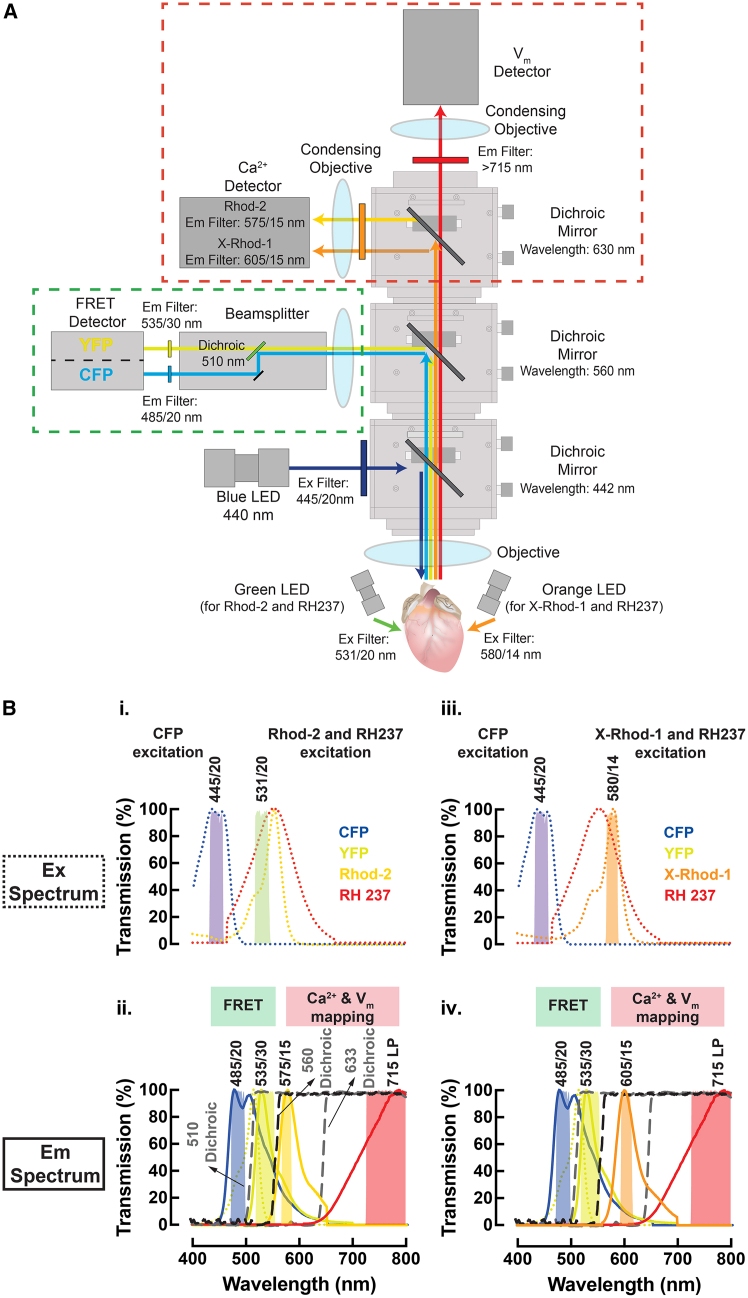
Quadruple-parametric FRET and dual optical mapping system (A) Schematic of the quadruple-parametric imaging system illustrating the optics used and the light path for cAMP FRET, *V*_m_, and Ca^2+^ signals. (B_i._), (B_iii._) Excitation and (B_ii._), (B_iv._) emission spectra of the three parameters—cAMP FRET (CFP and YFP), Rhod-2 or X-Rhod-1 (Ca^2+^), and RH237 (V_m_) fluorescence, illustrating the spectral separation of the three signals as implemented in this system. Dotted lines: excitation wavelength, solid lines: emission wavelengths, long-dotted vertical lines: dichroic mirrors, shaded boxes: filters.

A multiparametric whole-heart imaging system was built by modifying an existing optical mapping setup (THT Mesoscope, SciMedia; Figure 1A). Two light emitting diode (LED) excitation sources at 457 nm (Blue LED: T-LED+, Sutter Instrument) and 530 nm (Green LED: LEX-2, SciMedia) with collimators were used for epi-illumination of the heart to excite the FRET sensor and exogenous dyes, respectively. The emitted light signal is collected by a Planapo 1× lens (objective lens, WD = 61.5 mm, SciMedia) with infinity correction. The lens focuses the collected light at infinity and this correction allows multiple layers of filter sets and cameras to be introduced in the light path. The different wavelengths of the emitted light were separated via dichroic mirrors and filters, as depicted in Figure 1B, and then passed through the secondary Planapo 1X lens (projection lens, WD = 61.5 mm, SciMedia) prior to being captured by the cameras. The use of two lenses of the same focal length in tandem lens configuration results in a magnification of 1× (pixel size = 0.1 mm).^19^^,^^20^^,^^21^ The mapping system is equipped with one high-resolution sCMOS camera (Prime BSI, Photometrics) for FRET image acquisition and two high-speed CMOS cameras (MiCAM ULTIMA-L, SciMedia) for mapping *V*_m_ and Ca^2+^.

For FRET imaging, cyan fluorescent protein (CFP) is excited at 445/20 nm with the blue LED source (Figure 1B_i._) and CFP/yellow fluorescent protein (YFP) emission is reflected by the first long-pass dichroic at 560 nm (Figure 1B_ii._), collected by an image splitting device (OptoSplit II, Cairn Research, Ltd) and split onto a single sCMOS detector at 485/20 nm (CFP) and 535/30 nm (YFP) (Figure 1B_ii._). For dual optical mapping, RH237 (*V*_m_) and Rhod-2 (Ca^2+^) were excited at 531/20 nm with the green LED source (Figure 1B_i._). RH237 and Rhod-2 emission are transmitted by the first dichroic mirror, split by a second dichroic mirror at 633 nm (Figure 1B_ii._), and collected by two high-speed CMOS cameras at 715 nm/LP (RH237) and 575/15 nm (Rhod-2) (Figure 1B_ii._). The system also supports imaging of RH237 and the red-shifted Ca^2+^ indicator, X-Rhod-1, with 580 nm excitation (Figure 1B_iii._) and emission at 605/15 nm (Figure 1B_iv._). This dye combination will allow for greater spectral separation and simultaneous (rather than interleaved) excitation of the FRET fluorophore and exogenous dyes.

At the start of each experiment, a focusing target was positioned in front of the objective lens, and its position was adjusted until all three cameras were in clear focus. VisiView software (Visitron Systems GmbH) is utilized to control the sCMOS camera configuration, OptoSplit image alignment, and the blue LED on/off triggering. For the manual alignment of OptoSplit, VisiView facilitates the process by overlaying live images with contrasting colors, which effectively highlight misalignment. This real-time feedback enables rapid alignment of images through adjustment of the control knobs on the OptoSplit. BV Workbench (Brainvision, Inc.) serves as the software interface for the two high-speed CMOS cameras and the green LED on/off triggering. The CMOS cameras were spatially aligned by utilizing the Camera Calibration function within the software. This function superimposes images from different cameras and uses an edge detection algorithm, enabling manual adjustment of the angle of the dichroic mirrors until all fields of view are spatially aligned.

### Validation of FRET imaging

**Figure 2:**
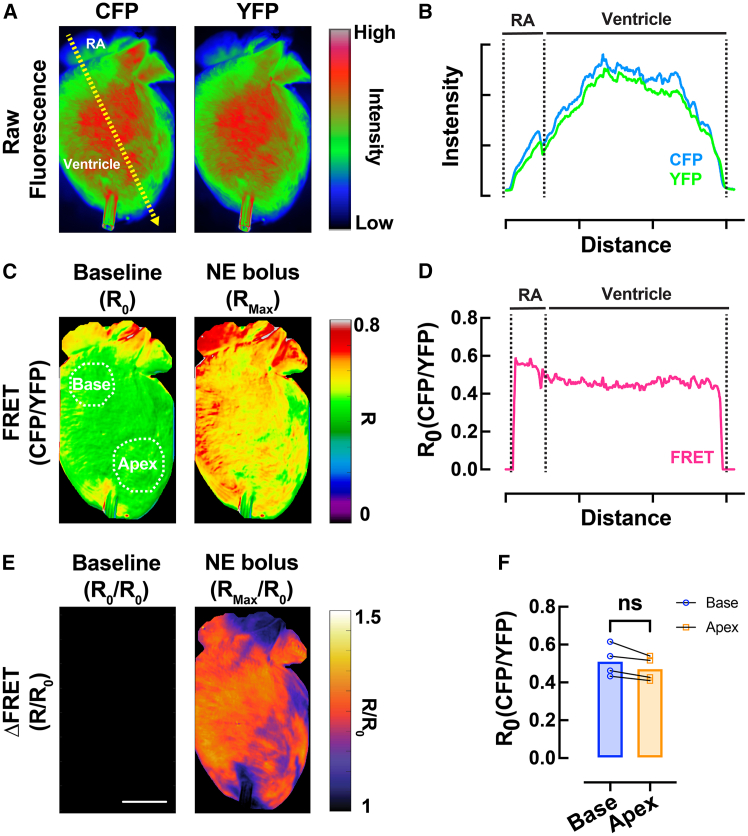
Validation of FRET imaging (A) Representative images of a cardiac-specific *CAMPER* mouse heart showing the raw intensity of CFP and YFP fluorescence upon CFP excitation. (B) Cross-sectional intensity of CFP and YFP from RA to apical left ventricle (yellow dashed arrow from A). (C) FRET maps (*R*, CFP/YFP) at baseline (*R*_0_) and during acute 1.5 μM NE bolus infusion. (D) Cross-sectional intensity of baseline FRET (*R*_0_) from RA to ventricle (yellow dashed arrow from A). (E) ΔFRET (*R*/*R*_0_) maps at baseline and during acute 1.5 μM NE bolus infusion. (F) Mean scatterplots from RV base (blue) and LV apex (orange) of *R*_0_. Paired *t* test, ∗*p* < 0.05, *N* = 4 hearts.

Langendorff-perfused hearts from *CAMPER* mice exhibited robust CFP and YFP fluorescence (Figure 2A), approximately 2- to 3-fold higher than the autofluorescence of isolated hearts from *CAMPER*/Cre^−^ controls.^22^ A representative cross-sectional intensity profile from the right atrium (RA) to the apical left ventricle is shown in Figure 2B, demonstrating weaker edge signal relative to the center across regions, which we attribute primarily to uneven illumination due to cardiac curvature rather than sensor expression gradients. Baseline FRET ratio maps (*R*_0_) demonstrated a stable spatial distribution (Figure 2C), with cross-sectional profiles confirming uniformity (Figure 2D) and no significant difference in *R*_0_ between the RV base and LV apex (Figure 2F). Upon acute β-AR stimulation with a norepinephrine bolus (NE, 1.5 μM), cAMP responses (FRET) became regionally heterogeneous across the heart (Figure 2C), as reflected in the ΔFRET maps (ΔFRET = *R*/*R*_0_; Figure 2E).

### Effects of blebbistatin on cAMP FRET signals

**Figure 3:**
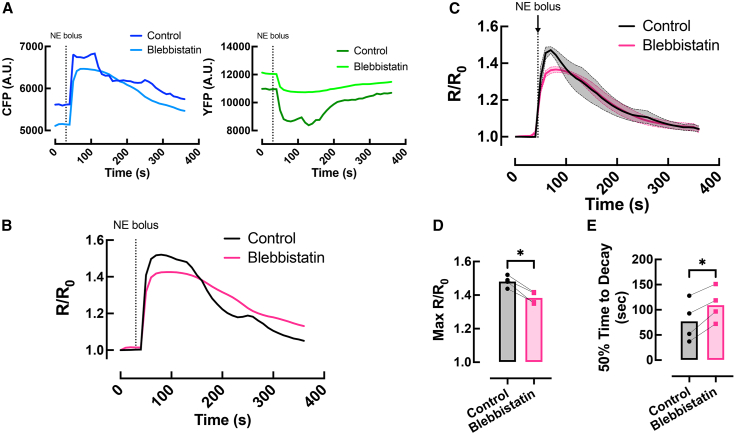
Effects of blebbistatin on cAMP FRET signals (A) Representative temporal CFP and YFP fluorescence. (B) ΔFRET response in a *CAMPER* mouse heart following application of a 1.5 μM bolus NE, recorded first without and then with 10 μM blebbistatin. (C) Average ΔFRET responses between hearts. (D) Max *R*/*R*_*0*_ and (E) 50% time to decay in response to bolus of 1.5 μM NE recorded first without and then with 10 μM blebbistatin. Paired *t* test, ∗*p* < 0.05, *N* = 4 hearts.

To determine whether the addition of the excitation-contraction uncoupler blebbistatin alters cAMP readouts, we infused hearts with acute NE (1.5 μM) first without and then with blebbistatin (10 μM). In the absence of blebbistatin, small inter-frame shifts were evident in the FRET signals due to contractile motion (Figures 3A and 3B); ensemble averaging between hearts reduced this motion-related variability (Figure 3C). Comparing conditions, the maximal cAMP response (*R*/*R*_0_) differed significantly, and the cAMP decay phase (reflecting phosphodiesterase-mediated cAMP breakdown) was significantly prolonged after blebbistatin. Given the need to minimize motion for high-speed optical mapping, all subsequent datasets were acquired after blebbistatin administration.

### Validation of spectral separation in multiparametric optical imaging

**Figure 4:**
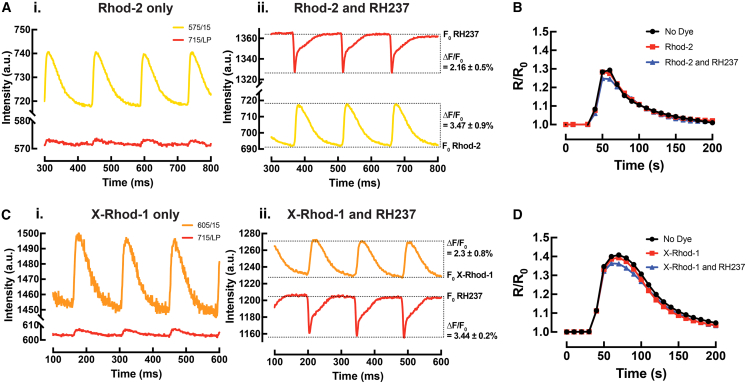
Validation of spectral separation (A_i._) Imaging of hearts stained with Rhod-2 or (C_i_) X-Rhod-1 only and (A_ii._) (C_ii_) following with RH237. (B) Representative FRET acquisition of example hearts stained with no dyes, Rhod-2 or (D) X-Rhod-1 only and with RH237 during an acute bolus of 0.5 μM NE infusion. Kolmogorov-Smirnov test, No dye vs. Rhod-2, *p* = 0.39; No dye vs. Both, *p* = 0.39; Rhod-2 vs. Both, *p* = 0.95. No dye vs. X-Rhod-1, *p* > 0.99; No dye vs. Both, *p* > 0.99; X-Rhod-1 vs. Both, *p* > 0.99, *N* = 3 hearts (Rhod-2); *N* = 1 heart (X-Rhod-1).

We then asked whether the exogenous voltage (RH237)- and calcium (Rhod-2 or X-Rhod-1)-sensitive dyes used for optical mapping would interfere with the cAMP FRET signal. To test for potential crosstalk between these dyes and the FRET fluorophores (CFP and YFP), experiments were performed on Langendorff-perfused hearts from cardiac-specific *CAMPER* mice. These hearts were initially stained with a single fluorescent dye (Rhod-2) and then subjected to interleaved FRET and dual optical mapping during an acute bolus of NE infusion (0.5 μM). Following this, the hearts were subsequently stained with RH237 and the same image acquisition protocol was repeated. The Kolmogorov-Smirnov test was performed to compare the similarity of FRET signal traces between different dye loadings. When hearts were stained with Rhod-2 alone, slight bleed-through of the Rhod-2 signal was observed in the *V*_m_ channel (Figure 4A_i._), but no change was observed in ΔFRET (*R*/*R*_0_) signal compared to hearts with no dye loading (Figure 4B). When hearts were stained with both Rhod-2 and RH237, the raw intensity of RH237 was high enough to strongly overwhelm the bleed-through of the Rhod-2 signal (Figure 4A_ii._), and there was no significant difference in FRET signal compared to hearts with no dye loading or with Rhod-2 only (Figure 4B). These results confirm spectral separation between *V*_m_, Ca^2+^, and FRET signals, and that loading of the exogenous dyes does not interfere with the cAMP FRET signal.

If simultaneous, rather than interleaved *V*_m_/Ca^2+^ and FRET imaging is desired, the red-shifted intracellular Ca^2+^ indicator (X-Rhod-1) in combination with RH237 can be used. The same dye loading and imaging acquisition protocol (as earlier) was repeated on Langendorff-perfused *CAMPER* mouse hearts. Similar to Rhod-2, the X-Rhod-1 signal exhibited slight bleed-through in the *V*_m_ channel, but this was negligible after RH237 loading (Figures 4C_i._ and 4C_ii._). No change was observed in FRET signal with any dye loading (Figure 4D).

### Spatiotemporal whole-heart cAMP and *V*_m_-Ca^2+^_*i*_ response following β-AR stimulation

**Figure 5:**
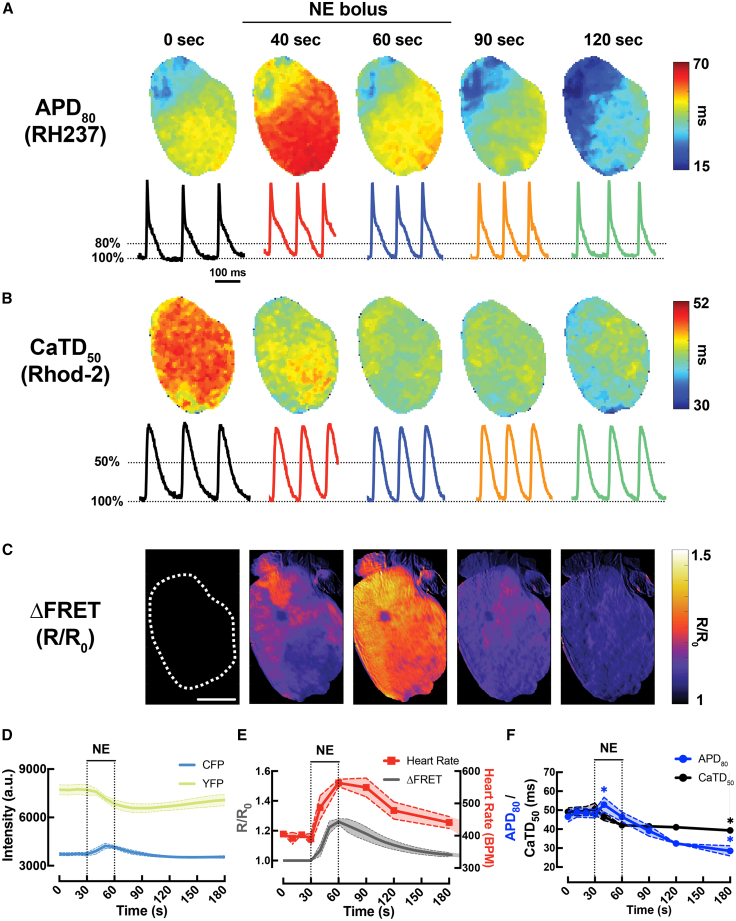
Demonstration of whole-heart cAMP and *V*_m_-Ca^2+^_*i*_ response following β-adrenergic stimulation (A) Example maps of action potential duration (APD_80_). (B) Calcium transient duration (CaTD_50_) and (C) ΔFRET images during acute 0.5 mM NE infusion (scale bars, 3 mm). (D) Temporal whole-heart CFP and YFP fluorescence responses in *CAMPER* mouse hearts after application of a 0.5 μM bolus NE. (E) Temporal whole-heart ΔFRET changes with heart rate and (F) whole-heart APD_80_, CaTD_50_ changes in response to 0.5 μM NE. Paired *t* test, ∗*p* < 0.05 vs. time = 0 s, *N* = 3 hearts. The same example heart, including APD_80_, CaTD_50_, and ΔFRET images, is shown in Figure 6 to demonstrate regional parameter quantification.

Next, we tested the capability of our quadruple-parametric optical mapping system to simultaneously capture cAMP FRET (CFP/YFP), *V*_m_ (RH237) and Ca^2+^_*i*_ (Rhod-2) activity during acute β-AR stimulation with a bolus of NE infusion (0.5 μM) in intact *CAMPER* hearts (*N* = 3). Maps of FRET, action potential duration (APD_80_), and calcium transient durationand calcium transient duration (CaTD_50_) during baseline (0 s), NE bolus (30–60 s) and washout (90–120 s) are shown in Figures 5A–5C. The application of an acute bolus of NE led to an increase in CFP fluorescence and a decrease in YFP fluorescence (Figure 5D), accompanied by a significant increase in both heart rate and the FRET ratio (Figure 5E). Similar to wild-type mouse hearts,^18^ the *CAMPER* hearts displayed a biphasic APD response (Figure 5F), where APD prolongation first occurred to maximum at ∼40 s of stimulation before shortening to near-baseline values by ∼60 s. Despite the biphasic APD response, CaTD showed a monotonic decrease in the *CAMPER* mouse hearts (Figure 5F).

**Figure 6:**
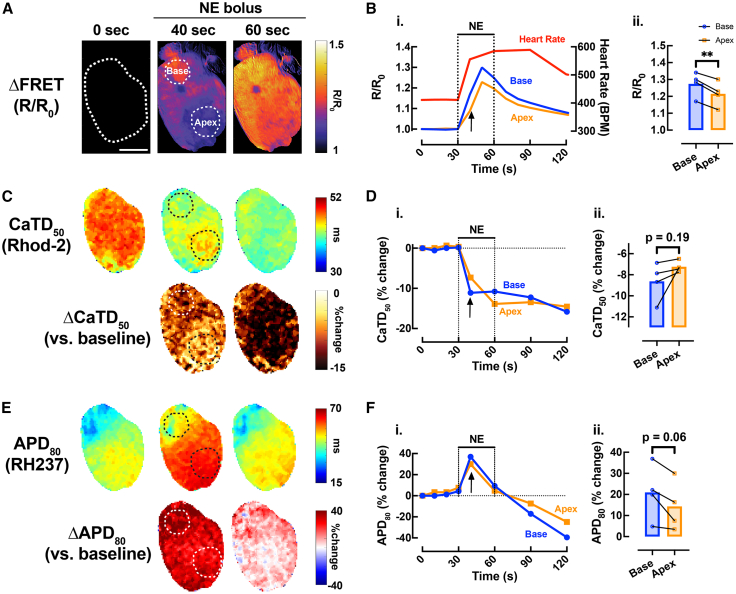
Regional effects of b-adrenergic stimulation on cAMP responsiveness in the *CAMPER* mouse heart (A) Representative ΔFRET ratio images from Figure 5C showing the spatiotemporal kinetics of cAMP activity (scale bars, 3 mm) and (B_i._) example FRET ratio trace and (B_ii._) mean scatterplots at time = 40 s (black arrow) in response to bolus of 0.5 μM NE from different regions of the heart (blue = RV base; orange = apex). (C) Representative CaTD_50_ (from Figure 5B) and (E) APD_80_ (from Figure 5A) maps from the heart in (A) (top) with change in CaTD_50_ and APD_80_ vs. baseline (ΔCaTD_50_ and ΔAPD_80_) (bottom). (D_i._) Example CaTD_50_ and (F_i._) APD_80_ percentage change trace over time and (D_ii._ and F_ii._) mean scatterplots from RV base (blue) and apex (orange) following bolus of 0.5 μM NE. Paired *t* test, ∗*p* < 0.05, *N* = 4 hearts.

To explore how spatiotemporal changes in cAMP signaling relate to electrophysiological responses, we analyzed two cardiac regions of interest (ROIs) from the RV base and apex. An example is shown in Figure 6. While cAMP levels followed a similar time course in both regions following β-AR stimulation, cAMP levels were elevated in the RV base compared to the apex during NE bolus infusion, with the most pronounced difference observed at 40 s (Figures 6A and 6B). Although not significantly different, we observed a trend toward greater shortening of CaTD_50_ and greater prolongation of APD_80_ in the RV base of the heart compared with the apex at the same time point (Figures 6C–6F).

### Real-time atrial cAMP and *V*_m_-Ca^2+^_*i*_ response following β-AR stimulation

**Figure 7:**
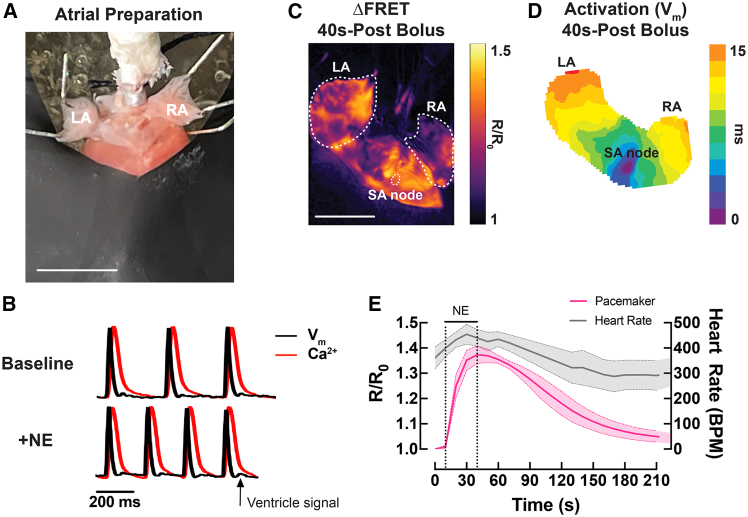
Atrial cAMP and *V*_m_-Ca^2+^_*i*_ response following β-adrenergic stimulation (A) Example atrial preparation for optical mapping in a Langendorff-perfused *CAMPER* heart. Left atrium = LA, right atrium = RA. Scale bars, 3 mm. (B) Representative *V*_m_ and Ca^2+^ optical traces in the atria before and after a bolus of 1.5 μM NE. Black arrow indicates slight ventricular signal bleed through due to light scattering. (C) Representative ΔFRET map and (D) *V*_m_ activation map in the atria after an acute bolus of 1.5 μM NE. Scale bars, 3 mm. (E) Averaged (mean = solid lines; SEM = shadow) temporal ΔFRET ratio traces in the sinoatrial node (SAN) with heart rate during bolus of 1.5 μM NE. *N* = 3 hearts.

To assess the capability of our optical imaging platform in visualizing pacemaker and atrial cAMP activity and *V*_m_-Ca^2+^_*i*_ responses, Langendorff-perfused *CAMPER* hearts were positioned in the posterior view and the ventricles were covered by a piece of flexible black plastic sheet to minimize ventricular light scattering to the atrial region (Figure 7A). Representative *V*_m_ and Ca^2+^_*i*_ traces recorded from the atria before and after an acute bolus of NE (1.5 μM) are shown in Figure 7B. Corresponding cAMP FRET and *V*_m_ activation maps recorded after an acute bolus of NE are shown in Figures 7C and 7D. The earliest site of activation within the intercaval region, identified from the *V*_m_ activation map, was used to functionally identify the leading pacemaker (sino-atrial node, SAN) in Figure 7D. This region was colocalized with the cAMP FRET ratio image (Figure 7C) to determine the cAMP response within the pacemaking region. The cAMP signal from the SAN region along with heart rate is plotted over time in Figure 7E.

### Whole-heart cAMP activity in response to sympathetic and parasympathetic stimulation

**Figure 8:**
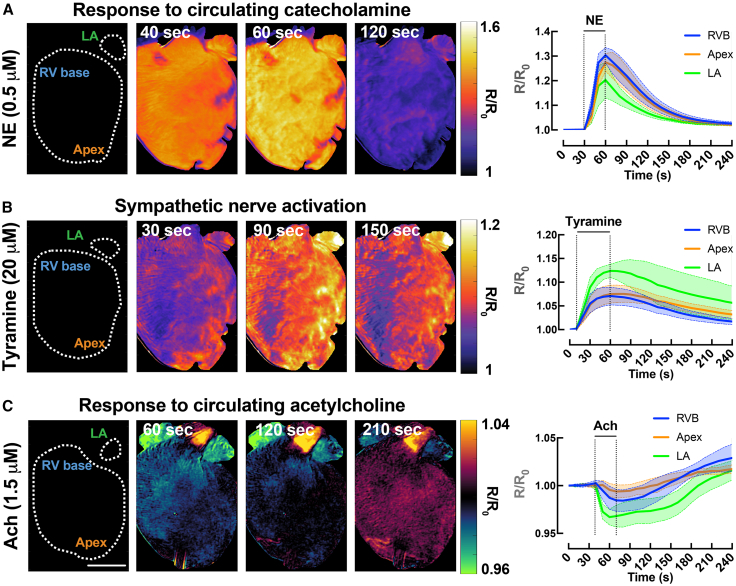
Effects of sympathetic and parasympathetic activation on whole-heart cAMP activity (A) Representative ΔFRET images showing the spatiotemporal kinetics of cAMP activity (scale bars = 3 mm) in response to bolus of 0.5 μM NE. (B) 20 μM tyramine and (C) 1.5 μM acetylcholine and averaged (mean = solid line; SEM = shadow) temporal ΔFRET traces from different regions of the heart (green = LA; blue = RV base; orange = apex). *N* = 3–4 hearts.

To assess whether the multiparametric system is capable of recording physiologically relevant autonomic modulation, we first compared whole-heart cAMP responses to exogenous NE versus chemically induced SNS. An example spatiotemporal cAMP response was assessed from the left atrium (LA), RV base and apex of the heart after NE bolus (0.5 μM). As can be observed in Figure 8A, exogenous stimulation with NE produced relatively similar cAMP magnitude and kinetics in the RV base vs. apex, but lower in LA. Chemical SNS with a bolus of tyramine (20 μM, tyramine causes release of NE from nerve terminals) to the heart was performed and the cAMP response is shown in Figure 8B. Chemical SNS showed a higher cAMP response in the LA compared to the RV base and apex, suggesting differences in NE release between the atrial and ventricular regions. To assess parasympathetic modulation, we applied acetylcholine (ACh, 1.5 μM) and measured whole-heart cAMP. As shown in Figure 8C, ACh (M2 receptor activation) produced a reversible reduction in cAMP with qualitative region-specific differences across the RV base, apex, and LA, demonstrating that the multiparametric system can resolve both sympathetic and parasympathetic influences on cAMP signaling.

## Discussion

In this study, we demonstrated and validated a multiparametric imaging system to capture real-time, whole-heart interactions between adrenergic signaling pathways and the corresponding functional responses during sympathetic and parasympathetic activation. Key findings include robust cAMP activation across the both atrial and ventricular regions in response to β-AR stimulation, synchronized with changes in heart rate, action potential, and calcium transient responses. Additionally, our mapping system demonstrated flexibility for imaging different cardiac regions, responses to both exogenous and endogenous catecholamines, cAMP reductions with parasympathetic activation, and integrating alternative calcium dyes to improve spectral separation and to support alternative fluorophore combinations. Building on these advancements, our approach offers the potential to expand multiparametric imaging toward other nodes of the β-AR signaling pathway. This is particularly relevant given recent advances in genetically encoded fluorescent biosensors, such as G-protein coupled receptor (GPCR) activation-based biosensors, which are widely used in a variety of neuroscience applications and could be used in cardiac signaling with similar precision.^23^^,^^24^ Such tools could significantly expand the scope of multi-parametric imaging, providing critical insights into the molecular mechanisms underlying cardiac dysfunction.

Our earlier research utilized a modified conventional cardiac optical mapping system to simultaneously measure cAMP signaling and *V*_m_ in the intact mouse heart. This study provided critical insights, revealing sex-dependent regional heterogeneity in cAMP breakdown and associated changes in APD and repolarization.^22^ Such findings underscore the complexity of adrenergic signaling pathways in cardiac function and their contribution to arrhythmogenic processes. While that study was the first to successfully image-specific molecular signaling and electrophysiological responses simultaneously at the whole-heart level, it did not address calcium dynamics. In the present study, we found regional variation in cAMP activity between the RV base and apex (Figure 6B_ii._), which corresponded to functional responses in both CaTD_50_ and APD_80_. Although these regional electrophysiological differences were not statistically significant, we observed a trend toward greater shortening of CaTD_50_ and prolongation of APD_80_ in the RV base of the heart compared with the apex (Figures 6D_ii._ and 6F_ii._).

Cardiac optical mapping of intracellular calcium dynamics and *V*_m_ is standard for studying excitation-contraction coupling and cardiac rhythms. However, it provides limited insight into specific molecular signaling events. In recent years, targeted biosensors have been used to monitor compartmentalized cAMP signaling in isolated cardiomyocytes, revealing the impact of receptor-microdomain disruptions in disease states.^25^ However, more recent studies have begun to assess cardiomyocyte signaling events within the intact heart. For example, Jungen et al. demonstrated how parasympathetic innervation regulates ventricular arrhythmia susceptibility via reductions in ventricular cAMP and shortening of refractory periods.^26^ Similarly, metabolic indicators, such as nicotinamide adenine dinucleotide (NADH), further highlight the need for integrative approaches. Variability in NADH fluctuations under stress conditions underscores differences between to Langendorff-perfused hearts and their responses to hypoxia, ischemia, or increased workload.^27^ These findings emphasize the utility of assessing signaling events in real-time in intact tissues and hearts.

Our group, along with others, are now pioneering efforts to combine traditional optical mapping with techniques that simultaneously reveal specific molecular signatures. This innovative approach bridges the gap between upstream molecular signaling and downstream electrophysiological outcomes, enabling a deeper understanding of the interplay between signaling, metabolism, excitation, and contraction in cardiac tissues. A notable advancement was recently reported by George et al., who used triple-parametric optical mapping to simultaneously measure Ca^2+^_*i*_, *V*_m_, and NADH, to investigate how metabolic state influences electrical and contractile behavior.^28^ Their findings highlighted how changes in metabolic activity influence electrophysiological responses and calcium handling, shedding light on the mechanisms underlying arrhythmogenesis and myocardial dysfunction in these settings. Future research should continue building on these advancements, focusing on the integration of additional molecular markers, and applying these methods to disease models, to enhance the scope of cardiac optical mapping and its translational potential.

### Limitations of the study

Mice expressing the cAMP biosensor *in vivo* were utilized in the study. However, mice have distinct differences in action potential kinetics and ion channel expression compared to humans,^29^ which may limit the translational relevance of the findings. When using NE to activate sympathetic responses, it is important to note that although its acute effects are largely β-AR/cAMP-PKA-mediated, NE also activates cAMP-independent pathways (e.g., α_1_-adrenergic/Gq signaling and effects on I_K1_), which can confound attribution of downstream functional effects, and should be interpreted with caution. The use of genetically encoded FRET biosensors, while powerful, also presents potential limitations, including influence on myocyte signaling networks. Similar to the mechanisms by which Ca^2+^-sensitive dyes may buffer intracellular Ca^2+^, intracellular cAMP buffering via binding to the Epac-based FRET sensor may interfere with endogenous cAMP dynamics. Additionally, expression levels of the FRET biosensor may vary between individual hearts and affect quantitative comparisons. To minimize motion artifacts during optical imaging, hearts were perfused with blebbistatin, a myosin II inhibitor that suppresses contraction. However, we found that blebbistatin modestly alters cAMP dynamics, which may be consistent with its known effects on cellular metabolism and adenosine triphosphate (ATP) availability.^30^ Indeed, elevated cellular ATP may enhance adenylyl cyclase substrate availability to sustain cAMP production during strong β-AR stimulation. Therefore, the mechanistic effects of blebbistatin on cAMP dynamics is an important area for future study. Finally, this study employed a novel multiparametric imaging system based on a tandem-lens epifluorescence macroscope setup. While this system offers substantial advantages in wide-field imaging, such as a 100–700 times brighter fluorescence image and improved light collection efficiency,^19^ it lacks the spatial and depth resolution of traditional high-resolution microscopy systems. The current configuration (∼7 μm per pixel for FRET imaging and ∼100 μm per pixel for *V*_m_/Ca^2+^ mapping) enables visualization of regional, tissue-level dynamics but not fine structural or subcellular signaling domains.

## Resource availability

### Lead contact

Requests for further information and resources should be directed to and will be fulfilled by the lead contact, Dr. Crystal M. Ripplinger (cripplinger@ucdavis.edu).

### Materials availability

• This study did not generate new unique reagents.
• Commercial stocks for the CAMPER floxed (no. 032205) and Myh6-Cre (no. 011038) mice are available via The Jackson Laboratory. Requests for the cardiac-specific CAMPER_CM_ line should be directed to the lead contact (distributed based on availability).

### Data and code availability

• Raw optical mapping images reported in this paper will be shared by the lead contact upon request.
• All original code is available in the supplemental information.
• Any additional information required to reanalyze the data reported in this paper is available from the lead contact upon request.

## Acknowledgments

This work was supported by grants from the National Institutes of Health (NIH) R01 HL111600, HL170626 (C.M.R), NIH K99 HL171836 (J.L.C.), and NIH T32 GM144303 (L.R.M). We thank Brady Okura, Toshi Sakuraba, and Kenji Tsubokura from SciMedia, USA for technical support. We thank Gianna Domeny for maintaining, breeding, and genotyping the cardiac-specific *CAMPER* mouse line.

## Author contributions

Conceptualization and supervision, C.M.R.; methodology, I.-J.E.L., J.L.C., and C.M.R.; data generation, I.-J.E.L., J.L.C., L.R.M., J.L.G., and L.W.; data analysis, I.-J.E.L., J.L.C., L.R.M., and J.L.G.; writing of the manuscript, I.-J.E.L. and C.M.R.; review and editing of the manuscript, I.-J.E. L., J.L.C., L.R.M., L.W., and C.M.R.

## Declaration of interests

The authors declare no competing interests.

## STAR★Methods

### Key resources table

**Table:** 

REAGENT or RESOURCE	SOURCE	IDENTIFIER
**Chemicals, peptides, and recombinant proteins**		

Sodium Chloride	Fisher Scientific	CAT# S271-500
Calcium Chloride Dihydrate	Sigma-Aldrich	CAT# 223506-500G
Potassium Chloride	Fisher Scientific	CAT# P217-500
Magnesium Chloride Hexahydrate	Sigma-Aldrich	CAT# 63064-500G
Sodium Bicarbonate	Sigma-Aldrich	CAT# S6297-1KG
Sodium Phosphate Monobasic Monohydrate	Fisher Scientific	CAT# S369-500
D-Glucose	Spectrum Chemical	CAT# TCI-G0048
Norepinephrine	Sigma-Aldrich	CAT# A0937-5G
Tyramine	Sigma-Aldrich	CAT# T2879-25G
Acetylcholine Chloride	Sigma-Aldrich	CAT# A9101-1VL
Blebbistatin	Tocris Bioscience	CAT# 1760
Pluronic F-127	Biotium	CAT# 59000
Rhod2-AM	Biotium	CAT# 50024
X-Rhod1-AM	Fisher Scientific	CAT# X14210
RH237	Biotium	CAT# 61018

**Experimental models: Organisms/strains**		

Mouse: C57BL/6-Gt(ROSA)26Sor^tm1(CAG-ECFP∗/Rapgef3/Venus∗)Kama^/J; (CAMPER floxed)	The Jackson Laboratory	JAX 032205
Mouse: B6.FVB-Tg(Myh6-cre)218Mds/J; (cardiac-specific alpha myosin-heavy chain (Myh6) Cre mouse)	The Jackson Laboratory	JAX 011038
Mouse: cardiac-specific CAMPER mice (CAMPER_CM_)	This paper	N/A

**Software and algorithms**		

MATLAB	MathWorks	https://www.mathworks.com; RRID:SCR_001622
Electromap	O’Shea et al.^31^	https://www.nature.com/articles/s41598-018-38263-2
FRET map	This paper	Supplemental file to this manuscript
ImageJ	National Institute of Health	https://imagej.nih.gov/ij/; RRID:SCR_003070
GraphPad Prism	GraphPad Software	https://www.graphpad.com; RRID:SCR_002798
VisiView software	Visitron Systems	https://www.visitron.de/products/visiviewr-software.html; RRID:SCR_022546
BV Workbench	SciMedia	https://www.scimedia.com/products/sw/bvwb4/

**Other**		

THT mesoscope	SciMedia	https://www.scimedia.com
Blue LED: T-LED+	Sutter Instrument	https://www.sutter.com/imaging/lambda-tled
Green LED: LEX-2	SciMedia	https://www.scimedia.com
Planapo 1X lens (objective and projection lens, WD = 61.5 mm)	SciMedia	https://www.scimedia.com
Prime BSI sCMOS camera	Photometrics	https://www.teledynevisionsolutions.com/products/prime-bsi/?vertical=tvs-photometrics&segment=tvs
MiCAM ULTIMA-L CMOS camera	SciMedia	https://www.scimedia.com
OptoSplit II	Cairn Research	https://cairn-research.co.uk/product/optosplit-ii/
Bandpass filter: 445/20, 531/20, 485/20, 535/30, 575/15, 715/LP, 580/14, 605/15	Semrock	https://www.idex-hs.com/semrock
Dichroic mirror: 442LP, 560LP, 510LP, 630LP	Semrock	https://www.idex-hs.com/semrock

### Experimental model and study participant details

#### Ethical approval

All procedures involving animals were approved by the Animal Care and Use Committee of the University of California, Davis (Reference No. 23850), and adhered to the Guide for the Care and Use of Laboratory Animals published by the National Institutes of Health (NIH Publication No. 85-23, revised 2011). Male (N = 9) and female (N = 4) CAMPER mice (bred in house at UC Davis) were house on a 12-h light-dark cycle and given access to food and water *ad libitum*.

#### Cardiac-specific CAMPER mice

Generation of cardiac-specific *CAMPER* mice was described previously.^22^ In brief, the *CAMPER* floxed mouse (Jackson Laboratories #032205), that reports cAMP binding by changes in FRET between donor (CFP) and acceptor (YFP) at the cAMP binding domain of Epac1,^32^ was crossed with the cardiac-specific alpha myosin-heavy chain (Myh6) Cre mouse (Jackson Laboratories #011038) for cardiac-specific expression. Mice were genotyped before use, and only homozygous male and female *CAMPER* mice 17 ± 5 weeks old were used for experiments.

### Method details

#### Whole-heart Langendorff perfusion

*CAMPER* mice received an intraperitoneal injection of heparin (100 IU) and were anesthetized with pentobarbital sodium (>150 mg/kg). For Langendorff perfusion, hearts were prepared according to previously established methods.^33^^,^^34^^,^^35^ In short, hearts were excised through a mid-sternal incision, placed in cold cardioplegia solution (with composition in mmol/L: NaCl 110, CaCl_2_ 1.2, KCl 16, MgCl_2_ 16, and NaHCO_3_ 10), and then cannulated at the ascending aorta. Subsequently, the hearts were retrograde perfused through the aorta with oxygenated modified Tyrode’s solution (in mM: NaCl 128.2, CaCl_2_ 1.3, KCl 4.7, MgCl_2_ 1.05, NaH_2_PO_4_ 1.19, NaHCO_3_ 20, and glucose 11.1; pH 7.4) at 37°C. The perfusion chamber was heated, and the perfusion pressure was maintained at 80 mmHg. To minimize motion artifacts, blebbistatin (10 μM, Tocris Bioscience) was added to the perfusate.^30^ Ag/AgCl needle electrodes were placed in the bath to record an electrocardiogram (ECG) similar to a lead I configuration.

#### FRET imaging and dual optical mapping

Hearts were equilibrated after cannulation and perfusion for 10 min followed by loading with calcium (Rhod-2 AM or X-Rhod-1 AM)-and voltage (RH237)-sensitive dyes. A 1 ml solution containing Rhod-2 AM (50 μl of 1 mg/ml stock solution mixed with 50 μl Pluronic F-127 and 900 μl Tyrode’s solution) or X-Rhod-1 AM (20 μl of 1 mg/ml stock solution mixed with 20 μl Pluronic F-127 and 960 μl Tyrode’s solution) was prepared and slowly injected into a port just proximal to the cannula with a 5 min dye washout period. Similarly, a 1 ml solution containing RH237 (5-10 μl of 5 mg/ml stock solution mixed with 970 μl Tyrode’s solution) was prepared and slowly injected into the dye port with a 5 min dye washout.

The *CAMPER* heart was then illuminated by two LED excitation light sources at wavelengths of 445/20 nm (blue) and 531/20 nm (green). While the former excites CFP, leading to YFP emission in the tissue, the latter excites both RH237 and Rhod-2 dyes. With this optical setup, there is spectral overlap between YFP emission and the green LED excitation. Therefore, interleaved imaging was performed to avoid signal crosstalk. CFP and YFP time-lapse imaging was acquired with a 100 ms exposure time every 10 s. Immediately following each CFP/YFP frame, the sCMOS camera sent out a TTL signal with a delay time to the CMOS cameras triggering V_m_ and Ca^2+^ recordings at a sampling rate of 1 kHz for 2 s. If simultaneous FRET and V_m_/Ca^2+^ imaging is required, RH237 and X-Rhod-1 can be used with LED excitation at 580/14 nm (orange). Since there is no spectral overlap between YFP emission and the orange LED excitation, FRET and V_m_/Ca^2+^ imaging can be acquired concurrently. CFP/YFP images were captured at a resolution of 1024 x 2048 pixels with a field of view of 7.24 mm x 14.5 mm (7 μm/pixel), while V_m_ and Ca^2+^ images were recorded at a resolution of 100 x 100 pixels with a field of view of 10 mm x 10 mm (100 μm/pixel).

Hearts were subjected to an acute bolus of norepinephrine (NE, 0.5 or 1.5 μM; Sigma-Aldrich), tyramine (20 μM; Sigma-Aldrich), or acetylcholine (1.5 μM; Sigma-Aldrich) to test responsiveness to β-adrenergic receptor (β-AR) or M2 muscarinic receptor (M2R) stimulation, respectively. Experiments were performed at 37°C under normal sinus rhythm, which allowed for assessment of heart rate changes and physiological activation and repolarization patterns.

### Quantification and statistical analysis

#### FRET and optical mapping data analysis

For whole-heart FRET analysis, a custom MATLAB (MathWorks) script was used to visualize cAMP responses. FRET was calculated as R, where R = donor/acceptor (CFP/YFP) and R_0_ = baseline CFP/YFP, on a pixel-by-pixel basis and visualized as a FRET map. To account for nonuniform illumination and heart curvature, FRET changes were expressed as ΔFRET = R/R_0_ and visualized as ΔFRET maps. ImageJ was used to define regions of interest (ROIs) manually at the right ventricular (RV) base and left ventricular (LV) apex, for which R/R_0_ was calculated. For each ROI, average FRET ratio time courses and responses at defined time points were plotted.

For V_m_ and Ca^2+^ data analysis, Electromap software was used as described previously.^31^ A binary mask of the epicardial surface was manually selected for whole-heart analysis. For post-processing of fluorescent signals, a spatial Gaussian filter (3 x 3 pixels) and a baseline drift correction (Top-Hat average) were used. Action potential (AP) duration (APD) at 80% (APD_80_) and Ca^2+^ transients duration (CaTD) at 50% (CaTD_50_) were computed as the time intervals from the activation time (time of maximum first derivative of the upstroke) to 80% of repolarization and 50% of calcium transient decay for 10 consecutive beats and averaged, respectively.^36^ Following APD and CaTD calculations, whole heart APD and CaTD image matrix maps were exported from Electromap. Fiducial and anatomical markers were used to align images, and ImageJ software was used to select the same ROIs as the FRET data, representing RV base and LV apex for APD_80_ and CaTD_50_. FRET ratio data were colocalized with APD and CaTD values for each ROI to determine the relationships between cAMP kinetics and functional outputs.

#### Statistical analysis

Data were analyzed in GraphPad Prism (version 10.6.1; GraphPad Software) and are presented as mean ± SEM, with N indicating the number of hearts. Regional differences were assessed using paired, two-tailed Student’s t-tests. Differences in the shapes of two signals were evaluated with the Kolmogorov–Smirnov test. Data were considered significant when p<0.05 (denoted by ∗ in figures).

## Supplemental information

Data S1. MATLAB script for ΔFRET map generation
