# Supplementary material for: Multiparametric imaging of spatio-temporal cAMP signaling, transmembrane potential, and intracellular calcium in the intact heart
Source: iScience. 2026 Jan 22;29(2):114779. doi: 10.1016/j.isci.2026.114779 (PMC12915281; doi:10.1016/j.isci.2026.114779)
Supplement: Data S1. MATLAB script for ΔFRET map generation [file mmc1.zip › Matlab script/natsortfiles/html/natsortfiles_doc.html]

NATSORTFILES Examples 

# NATSORTFILES Examples

The function NATSORTFILES sorts a cell array of filenames or filepaths (1xN char), taking into account any number values within the strings. This is known as a *natural order sort* or an *alphanumeric sort*. Note that MATLAB's inbuilt SORT function sorts the character codes only (as does SORT in most programming languages).

NATSORTFILES is not a naive natural-order sort, but splits and sorts filenames and file extensions separately, which means that NATSORTFILES sorts shorter filenames before longer ones: this is known as a *dictionary sort*. For the same reason filepaths are split at every path-separator character (either '\' or '/'), and each directory level is sorted separately. See the "Explanation" sections below for more details.

For sorting the rows of a cell array of strings use NATSORTROWS.

For sorting a cell array of strings use NATSORT.

## Contents

- Basic Usage
- Output 2: Sort Index
- Output 3: Debugging Array
- Input 2: Regular Expression
- Inputs 3+: Optional Arguments
- Example with DIR and a Cell Array
- Example with DIR and a Structure
- Explanation: Dictionary Sort
- Explanation: Filenames
- Explanation: Filepaths
- Regular Expression: Decimal Numbers, E-notation, +/- Sign
- Bonus: Interactive Regular Expression Tool

## Basic Usage

By default NATSORTFILES interprets consecutive digits as being part of a single integer, any remaining substring/s are treated as characters:

```
A = {'a2.txt', 'a10.txt', 'a1.txt'};
sort(A)
natsortfiles(A)
```

```
ans = 
    'a1.txt'    'a10.txt'    'a2.txt'
ans = 
    'a1.txt'    'a2.txt'    'a10.txt'
```

## Output 2: Sort Index

The second output argument is a numeric array of the sort indices ndx, such that Y = X(ndx) where Y = natsortfiles(X):

```
[~,ndx] = natsortfiles(A)
```

```
ndx =
     3     1     2
```

## Output 3: Debugging Array

The third output is a cell vector of cell arrays, where the cell arrays correspond to the directory hierarchy, filenames, and file extensions. The cell arrays contain all matched numbers (after converting to numeric using the specified SSCANF format) and all split character substrings. These cell arrays are useful for confirming that the numbers are being correctly identified by the regular expression. Note that the even columns contain any matched number values, while the odd columns contain any split substrings:

```
[~,~,dbg] = natsortfiles(A);
dbg{:}
```

```
ans = 
    'a'    [ 2]
    'a'    [10]
    'a'    [ 1]
ans = 
    '.txt'
    '.txt'
    '.txt'
```

## Input 2: Regular Expression

The optional second input argument is a regular expression which specifies the number matching:

```
B = {'1.3.txt','1.10.txt','1.2.txt'};
natsortfiles(B)   % by default match integers
natsortfiles(B, '\d+\.?\d*') % match decimal fractions
```

```
ans = 
    '1.2.txt'    '1.3.txt'    '1.10.txt'
ans = 
    '1.10.txt'    '1.2.txt'    '1.3.txt'
```

## Inputs 3+: Optional Arguments

Further inputs are passed directly to NATSORT, thus giving control over the case sensitivity, sort direction, and other options. See the NATSORT help for explanations and examples of the supported options:

```
C = {'B.txt','10.txt','1.txt','A.txt','2.txt'};
natsortfiles(C, [], 'descend')
natsortfiles(C, [], 'char<num')
```

```
ans = 
    'B.txt'    'A.txt'    '10.txt'    '2.txt'    '1.txt'
ans = 
    'A.txt'    'B.txt'    '1.txt'    '2.txt'    '10.txt'
```

## Example with DIR and a Cell Array

One common situation is to use DIR to identify files in a folder, sort them into the correct order, and then loop over them: below is an example of how to do this. Remember to preallocate all output arrays before the loop!

```
P = 'natsortfiles_test'; % directory path
S = dir(fullfile(P,'*.txt')); % get list of files in directory
C = natsortfiles({S.name}); % sort file names into order
for k = 1:numel(C)
    disp(fullfile(P,C{k}))
end
```

```
natsortfiles_test\A_1.txt
natsortfiles_test\A_1-new.txt
natsortfiles_test\A_1_new.txt
natsortfiles_test\A_2.txt
natsortfiles_test\A_3.txt
natsortfiles_test\A_10.txt
natsortfiles_test\A_100.txt
natsortfiles_test\A_200.txt
```

## Example with DIR and a Structure

Users who need to access the DIR structure fields can use NATSORTFILE's second output to sort DIR's output structure into the correct order:

```
P = 'natsortfiles_test'; % directory path
S = dir(fullfile(P,'*.txt')); % get list of files in directory
[~,ndx] = natsortfiles({S.name}); % indices of correct order
S = S(ndx); % sort structure using indices
for k = 1:numel(S)
    fprintf('%-13s%s\n',S(k).name,S(k).date)
end
```

```
A_1.txt      22-Jul-2017 09:13:24
A_1-new.txt  22-Jul-2017 09:13:24
A_1_new.txt  22-Jul-2017 09:13:24
A_2.txt      22-Jul-2017 09:13:24
A_3.txt      22-Jul-2017 09:13:24
A_10.txt     22-Jul-2017 09:13:24
A_100.txt    22-Jul-2017 09:13:24
A_200.txt    22-Jul-2017 09:13:24
```

## Explanation: Dictionary Sort

Filenames and file extensions are separated by the extension separator: the period character '.'. Using a normal SORT the period gets sorted *after* all of the characters from 0 to 45 (including !"#$%&'()\*+,-, the space character, and all of the control characters, e.g. newlines, tabs, etc). This means that a naive natural-order sort will sort some short filenames after longer filenames. In order to provide the correct dictionary sort, with shorter filenames first, NATSORTFILES splits filenames from file extensions and sorts them separately:

```
D = {'test_ccc.m'; 'test-aaa.m'; 'test.m'; 'test.bbb.m'};
sort(D) % '-' sorts before '.'
natsort(D) % '-' sorts before '.'
natsortfiles(D) % correct dictionary sort
```

```
ans = 
    'test-aaa.m'
    'test.bbb.m'
    'test.m'
    'test_ccc.m'
ans = 
    'test-aaa.m'
    'test.bbb.m'
    'test.m'
    'test_ccc.m'
ans = 
    'test.m'
    'test-aaa.m'
    'test.bbb.m'
    'test_ccc.m'
```

## Explanation: Filenames

NATSORTFILES combines a dictionary sort with a natural-order sort, so that the number values within the filenames are taken into consideration:

```
E = {'test2.m'; 'test10-old.m'; 'test.m'; 'test10.m'; 'test1.m'};
sort(E) % Wrong numeric order.
natsort(E) % Correct numeric order, but longer before shorter.
natsortfiles(E) % Correct numeric order and dictionary sort.
```

```
ans = 
    'test.m'
    'test1.m'
    'test10-old.m'
    'test10.m'
    'test2.m'
ans = 
    'test1.m'
    'test2.m'
    'test10-old.m'
    'test10.m'
    'test.m'
ans = 
    'test.m'
    'test1.m'
    'test2.m'
    'test10.m'
    'test10-old.m'
```

## Explanation: Filepaths

For the same reason, filepaths are split at each file path separator character (both '/' and '\' are treated as file path separators) and every level of the directory structure are sorted separately. This ensures that the directory names are sorted with a dictionary sort and that any numbers are taken into consideration:

```
F = {'A2-old\test.m';'A10\test.m';'A2\test.m';'AXarchive.zip';'A1\test.m'};
sort(F) % Wrong numeric order, and '-' sorts before '\':
natsort(F) % correct numeric order, but longer before shorter.
natsortfiles(F) % correct numeric order and dictionary sort.
```

```
ans = 
    'A10\test.m'
    'A1\test.m'
    'A2-old\test.m'
    'A2\test.m'
    'AXarchive.zip'
ans = 
    'A1\test.m'
    'A2-old\test.m'
    'A2\test.m'
    'A10\test.m'
    'AXarchive.zip'
ans = 
    'AXarchive.zip'
    'A1\test.m'
    'A2\test.m'
    'A2-old\test.m'
    'A10\test.m'
```

## Regular Expression: Decimal Numbers, E-notation, +/- Sign

NATSORTFILES number matching can be customized to detect numbers with a decimal fraction, E-notation, a +/- sign, binary/hexadecimal, or other required features. The number matching is specified using an appropriate regular expression: see NATSORT for details and examples.

```
G = {'1.23V.csv','-1V.csv','+1.csv','+NaNV.csv','1.200V.csv'};
natsortfiles(G) % by default match integers
natsortfiles(G,'[-+]?(NaN|Inf|\d+\.?\d*)')
```

```
ans = 
    '1.23V.csv'    '1.200V.csv'    '+1.csv'    '+NaNV.csv'    '-1V.csv'
ans = 
    '-1V.csv'    '+1.csv'    '1.200V.csv'    '1.23V.csv'    '+NaNV.csv'
```

## Bonus: Interactive Regular Expression Tool

Regular expressions are powerful and compact, but getting them right is not always easy. One assistance is to download my interactive tool IREGEXP, which lets you quickly try different regular expressions and see all of REGEXP's outputs displayed and updated as you type.

Published with MATLAB® R2012b
