# Supplementary figures and images for: Multiparametric imaging of spatio-temporal cAMP signaling, transmembrane potential, and intracellular calcium in the intact heart
Source: iScience. 2026 Jan 22;29(2):114779. doi: 10.1016/j.isci.2026.114779 (PMC12915281; doi:10.1016/j.isci.2026.114779)

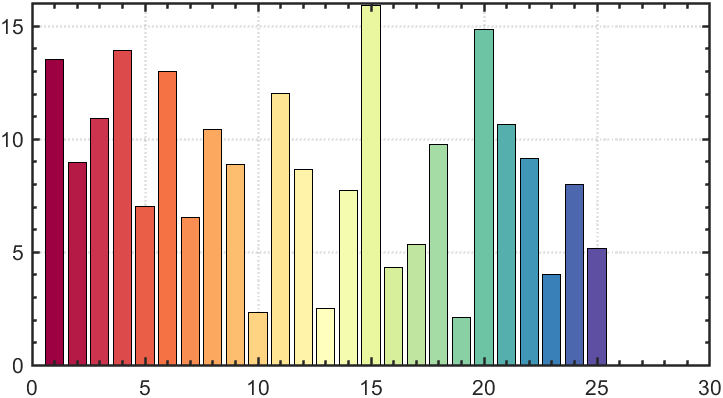

Supplement: Data S1. MATLAB script for ΔFRET map generation [file mmc1.zip › Matlab script/slanCM/slanCM/_demo7_4.png]

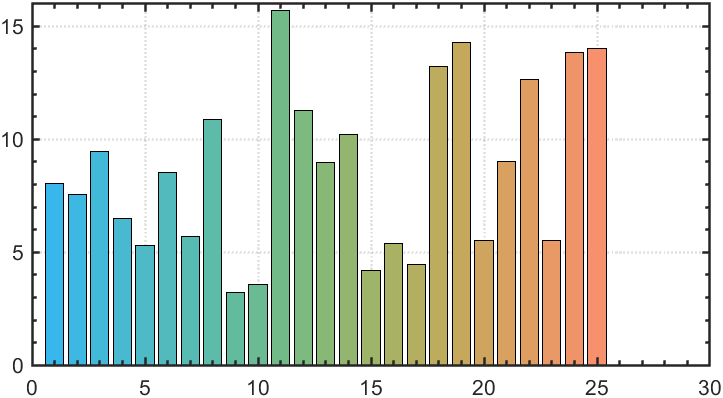

Supplement: Data S1. MATLAB script for ΔFRET map generation [file mmc1.zip › Matlab script/slanCM/slanCM/_demo7_5.png]

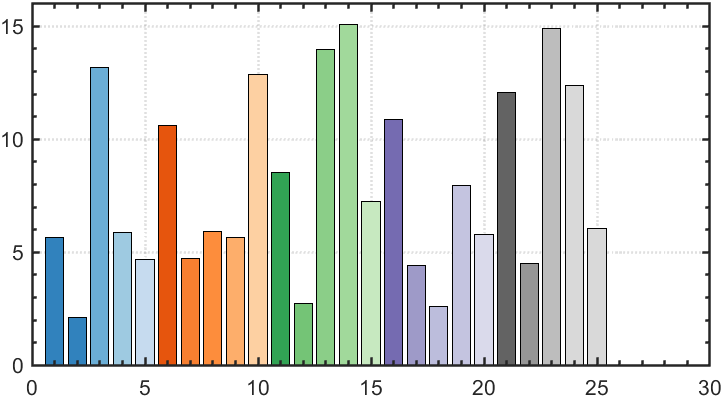

Supplement: Data S1. MATLAB script for ΔFRET map generation [file mmc1.zip › Matlab script/slanCM/slanCM/_demo7_7.png]

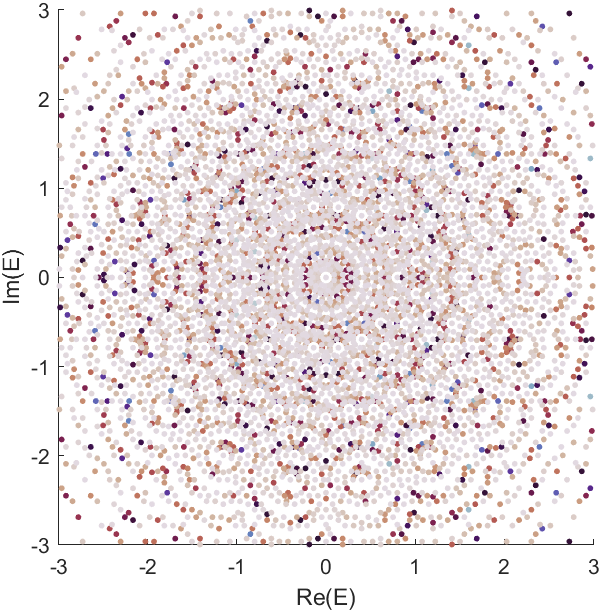

Supplement: Data S1. MATLAB script for ΔFRET map generation [file mmc1.zip › Matlab script/slanCM/slanCM/_demo8.png]

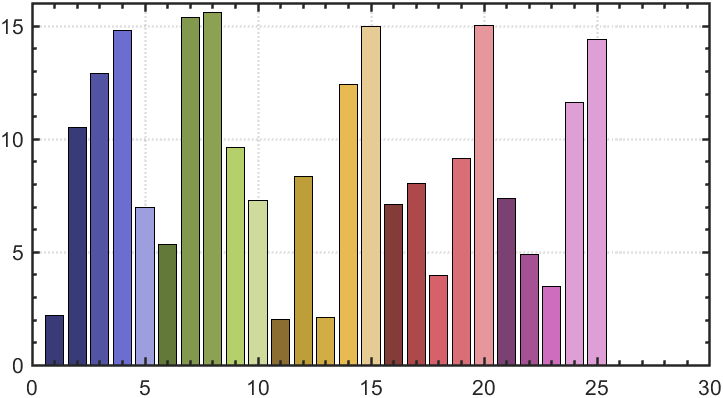

Supplement: Data S1. MATLAB script for ΔFRET map generation [file mmc1.zip › Matlab script/slanCM/slanCM/_demo7_6.png]

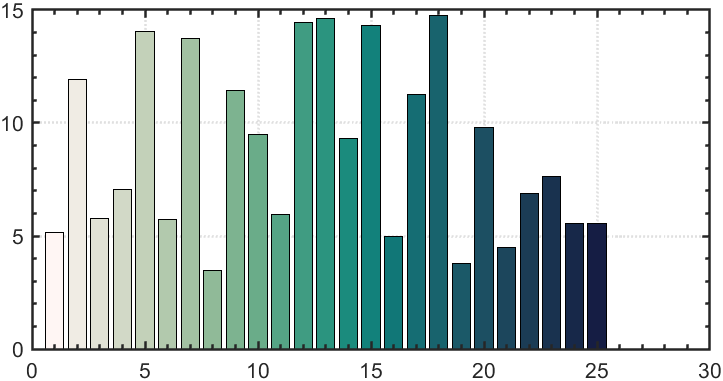

Supplement: Data S1. MATLAB script for ΔFRET map generation [file mmc1.zip › Matlab script/slanCM/slanCM/_demo7_2.png]

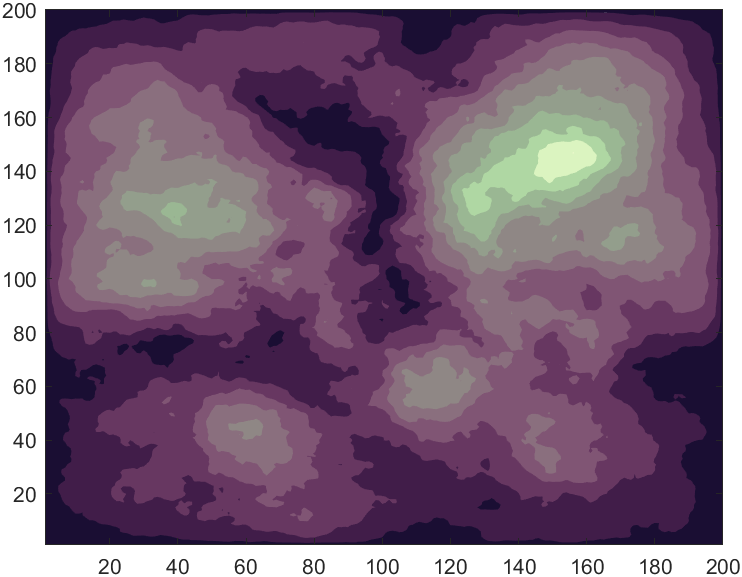

Supplement: Data S1. MATLAB script for ΔFRET map generation [file mmc1.zip › Matlab script/slanCM/slanCM/_demo5_1.png]

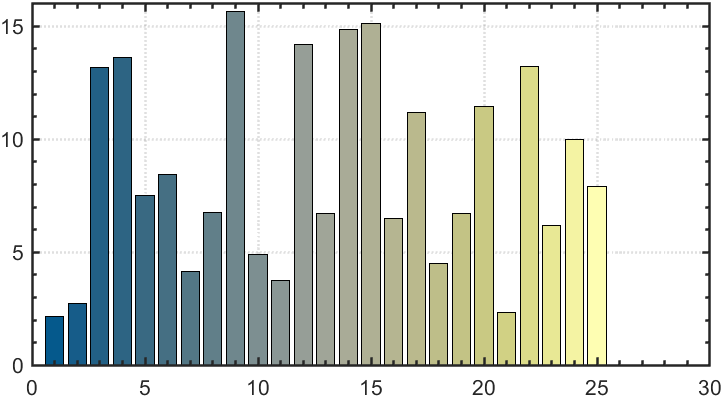

Supplement: Data S1. MATLAB script for ΔFRET map generation [file mmc1.zip › Matlab script/slanCM/slanCM/_demo7_3.png]

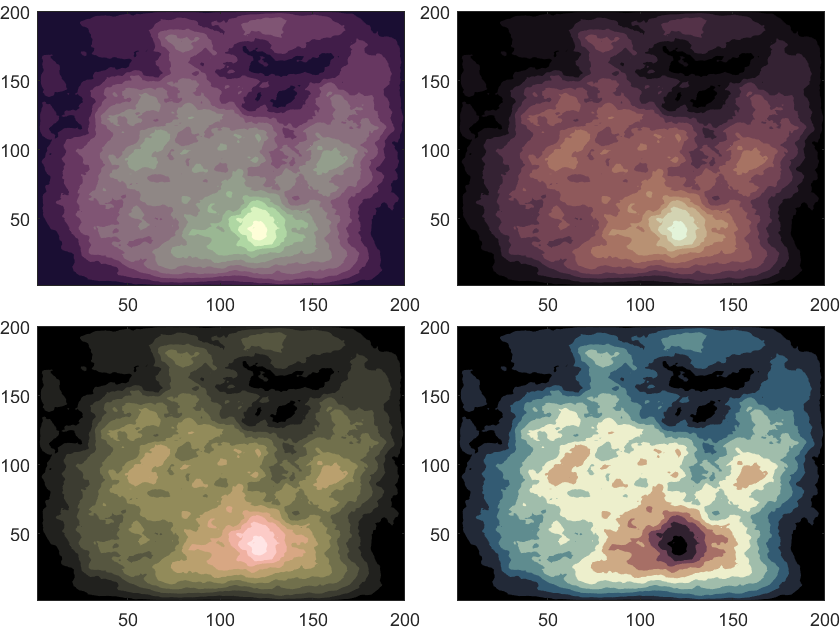

Supplement: Data S1. MATLAB script for ΔFRET map generation [file mmc1.zip › Matlab script/slanCM/slanCM/_demo5_2.png]

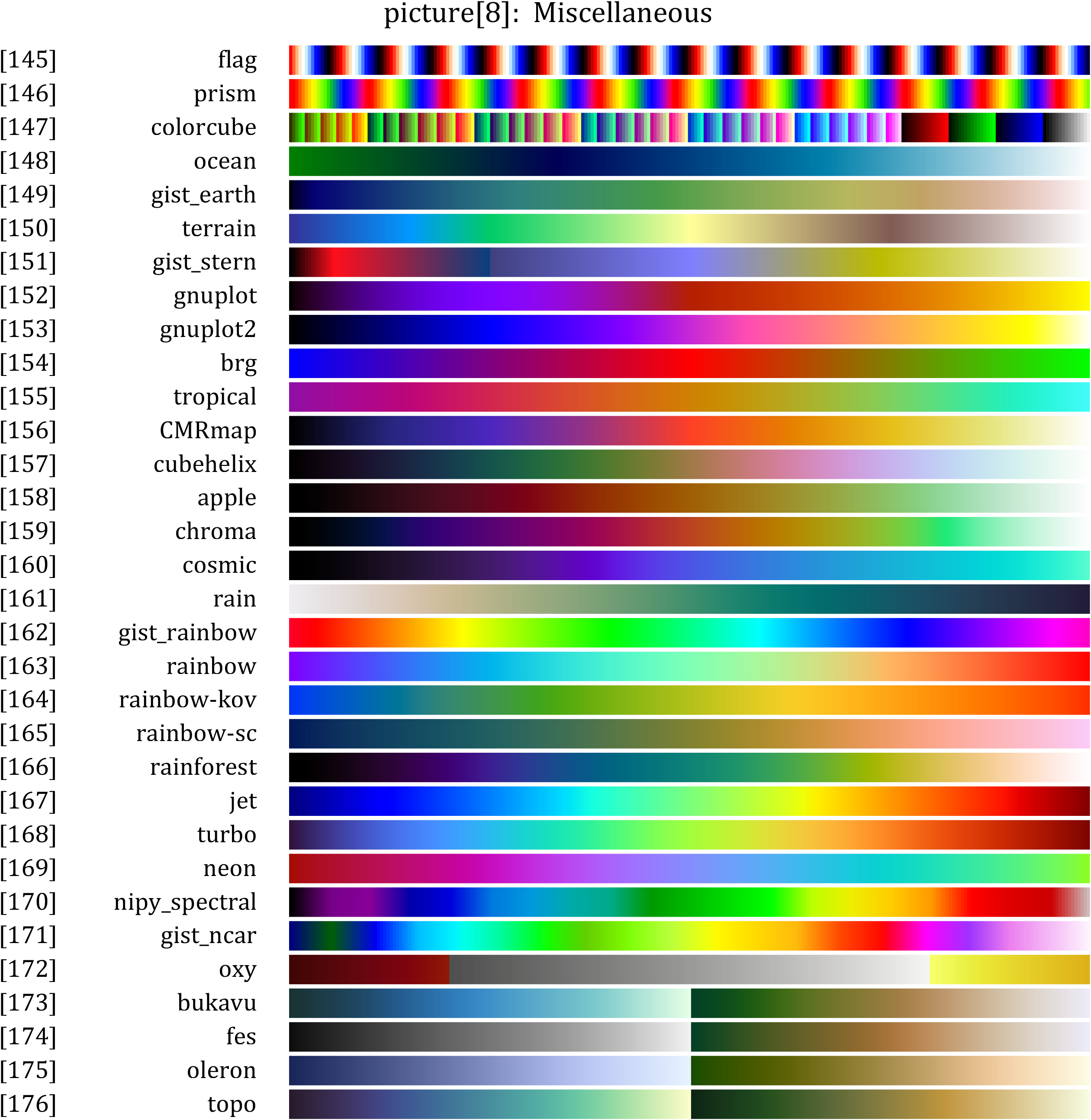

Supplement: Data S1. MATLAB script for ΔFRET map generation [file mmc1.zip › Matlab script/slanCM/slanCM/_8.png]

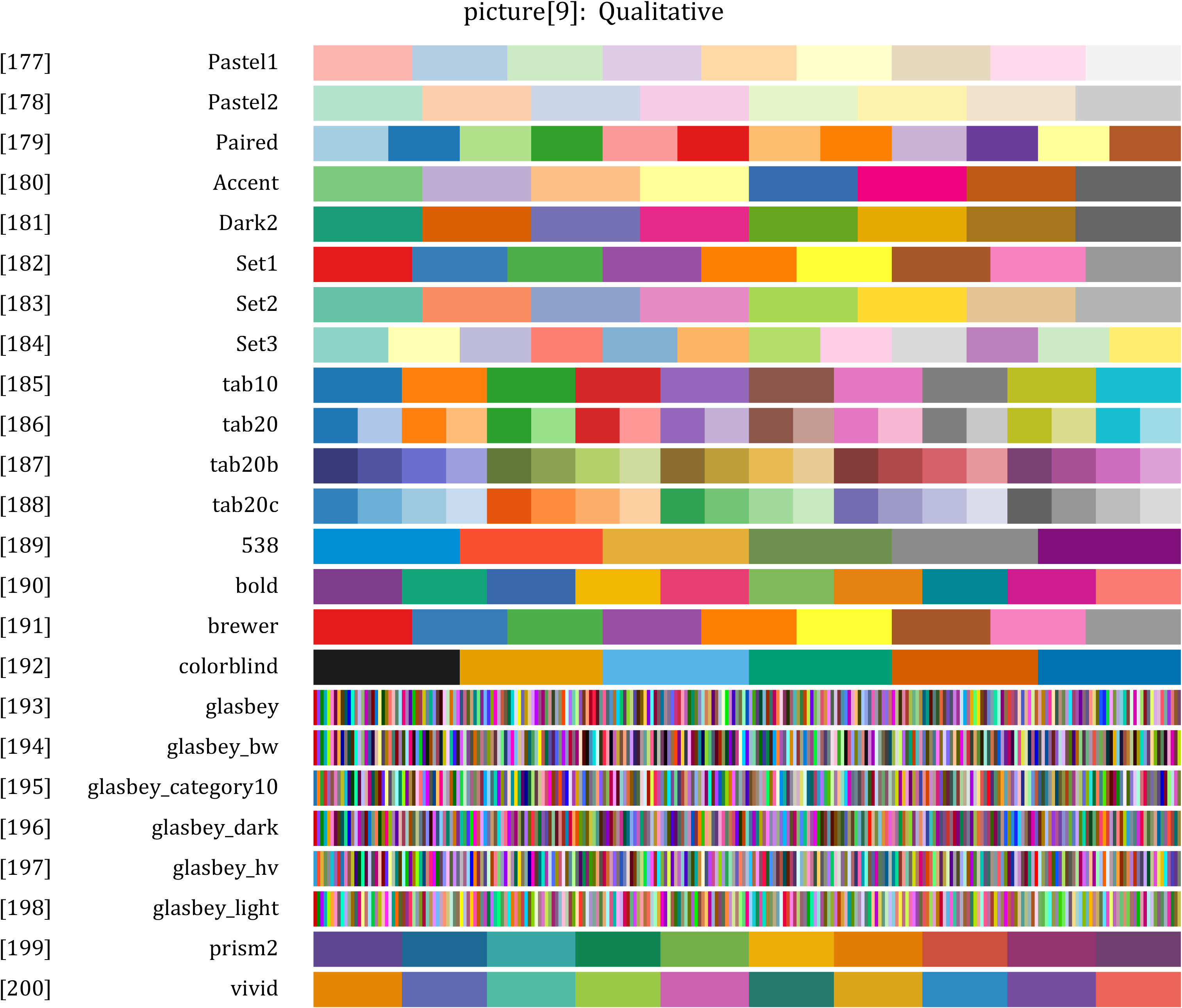

Supplement: Data S1. MATLAB script for ΔFRET map generation [file mmc1.zip › Matlab script/slanCM/slanCM/_9.png]

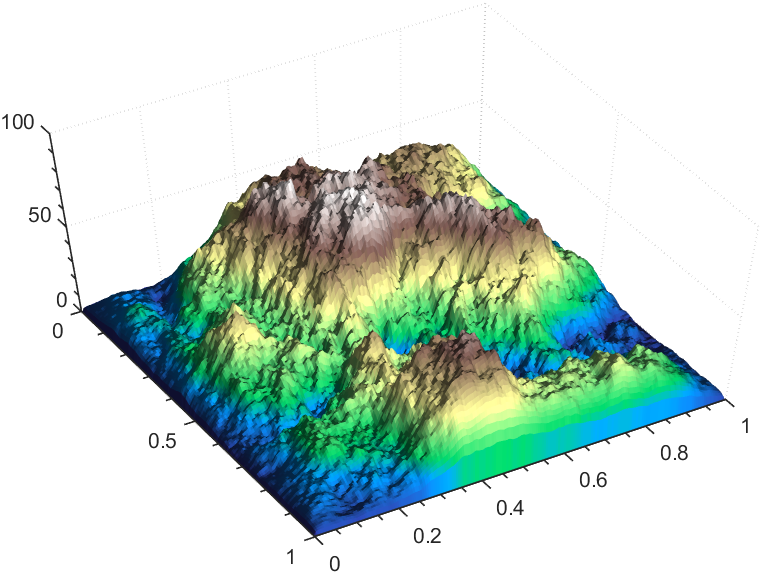

Supplement: Data S1. MATLAB script for ΔFRET map generation [file mmc1.zip › Matlab script/slanCM/slanCM/_demo4_2.png]

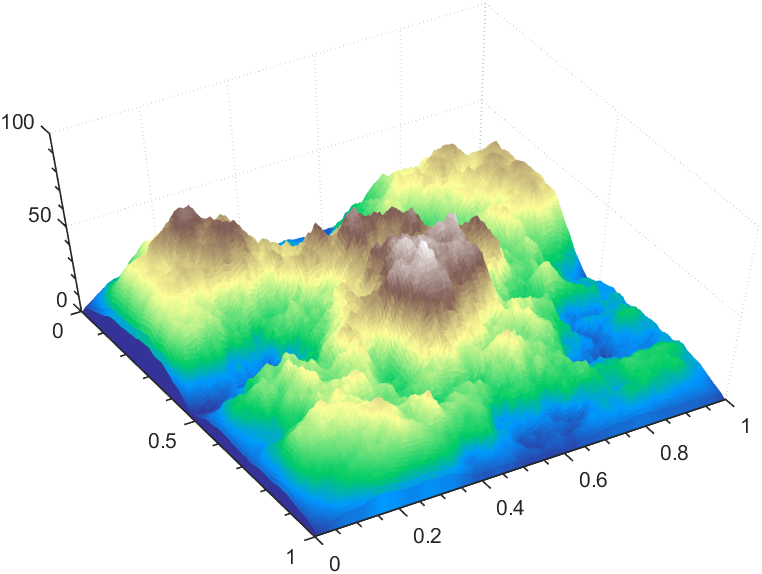

Supplement: Data S1. MATLAB script for ΔFRET map generation [file mmc1.zip › Matlab script/slanCM/slanCM/_demo4_1.png]

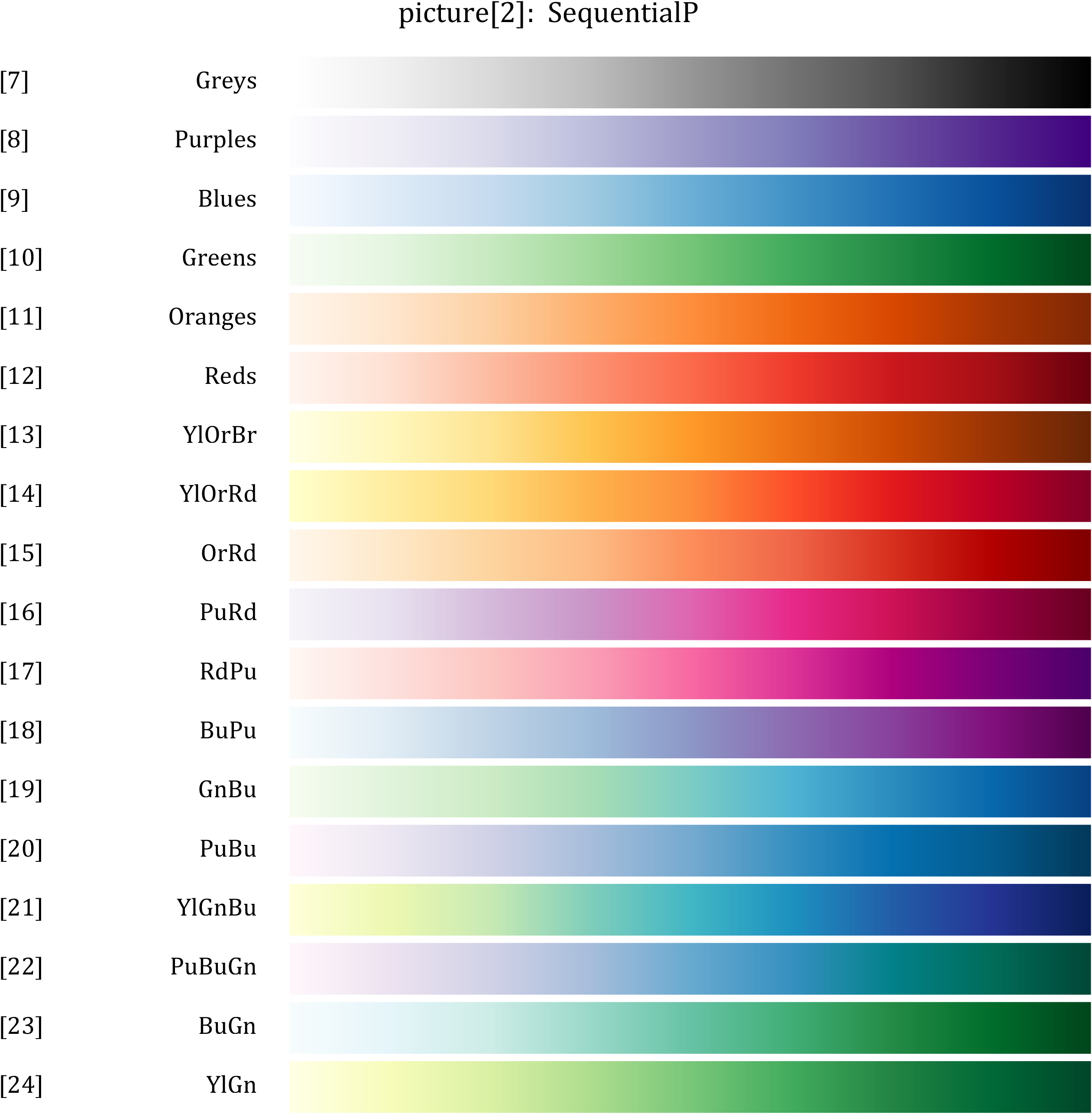

Supplement: Data S1. MATLAB script for ΔFRET map generation [file mmc1.zip › Matlab script/slanCM/slanCM/_2.png]

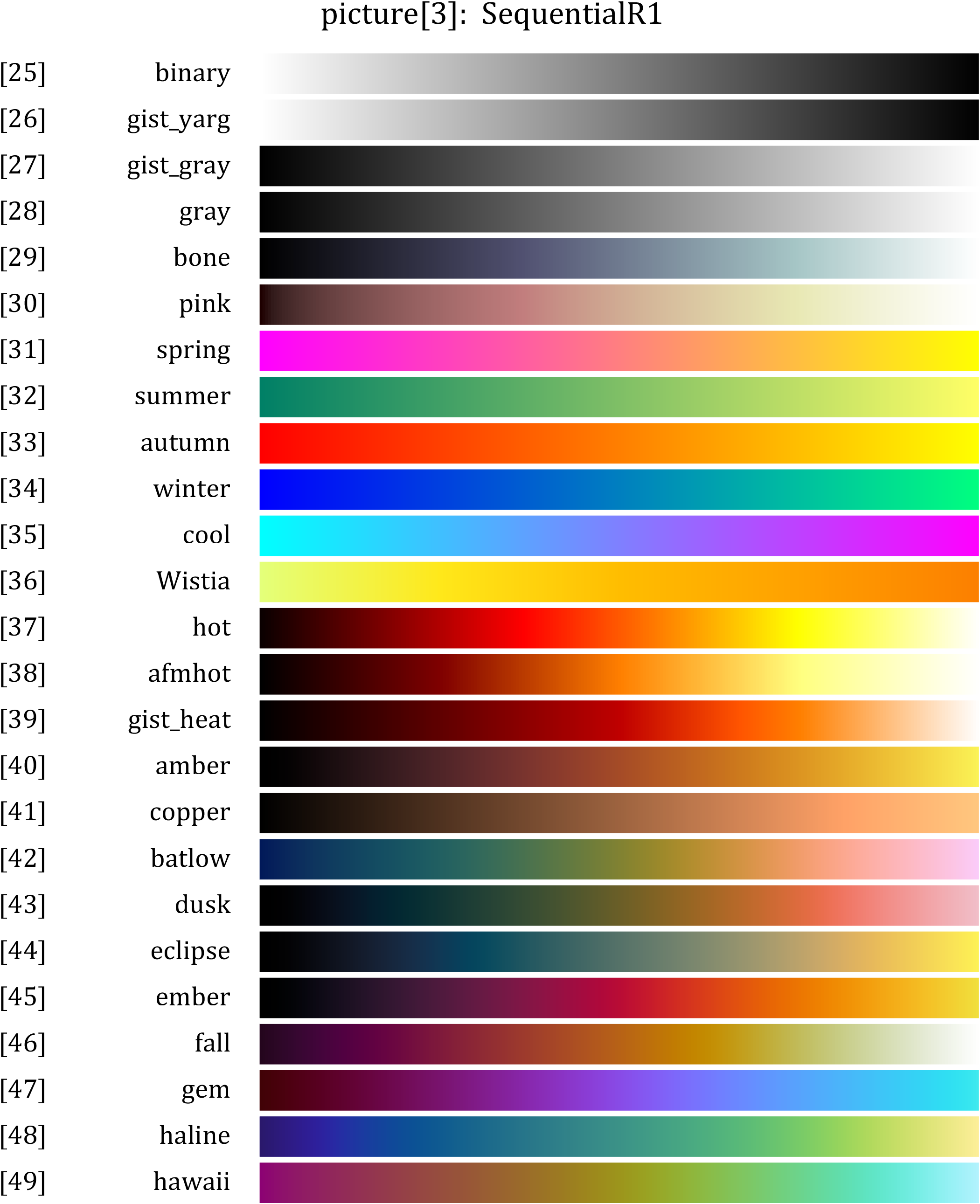

Supplement: Data S1. MATLAB script for ΔFRET map generation [file mmc1.zip › Matlab script/slanCM/slanCM/_3.png]

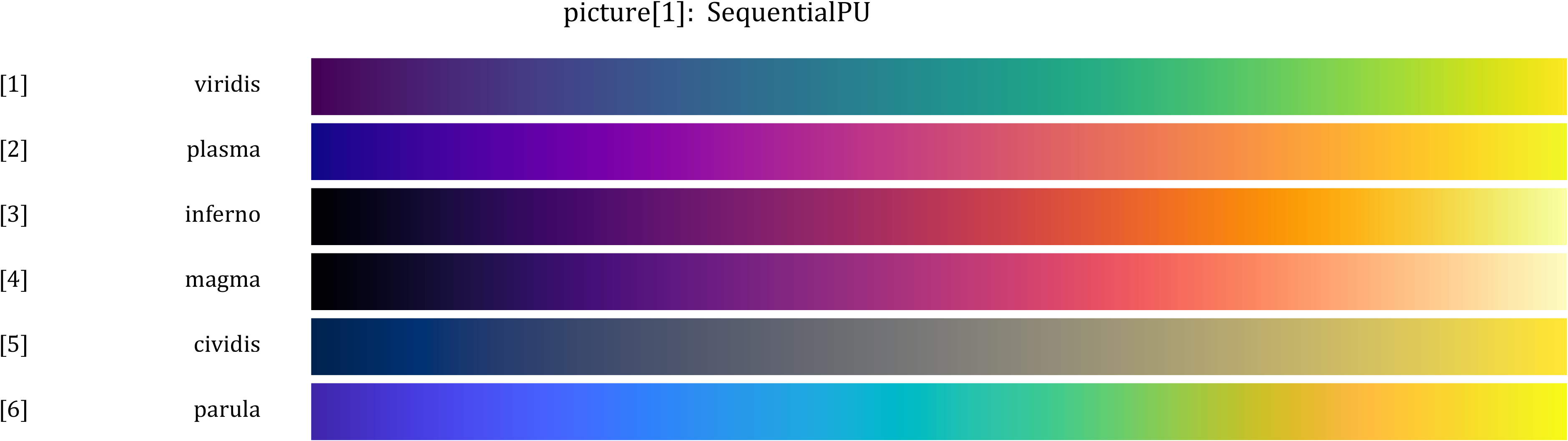

Supplement: Data S1. MATLAB script for ΔFRET map generation [file mmc1.zip › Matlab script/slanCM/slanCM/_1.png]

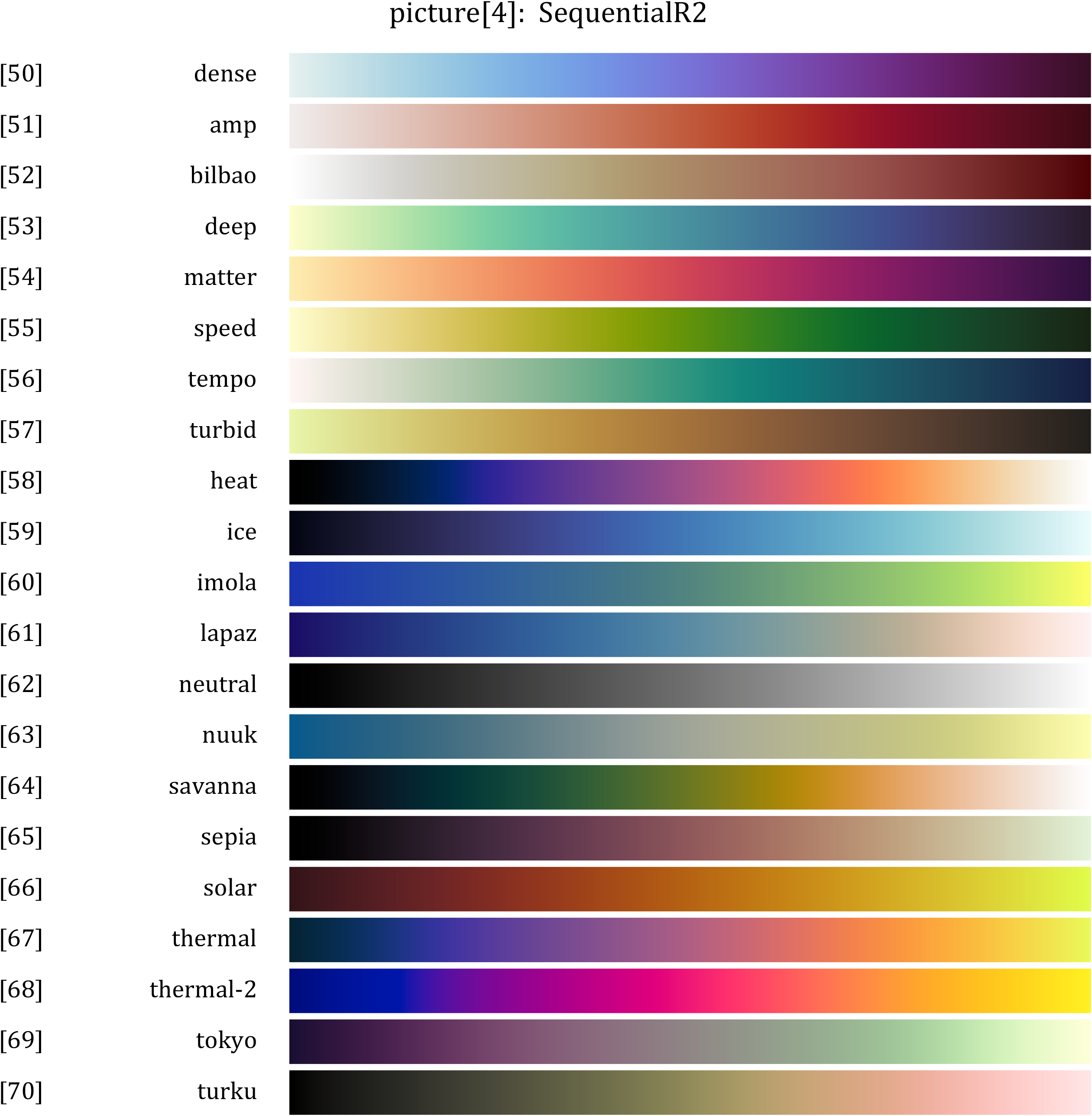

Supplement: Data S1. MATLAB script for ΔFRET map generation [file mmc1.zip › Matlab script/slanCM/slanCM/_4.png]

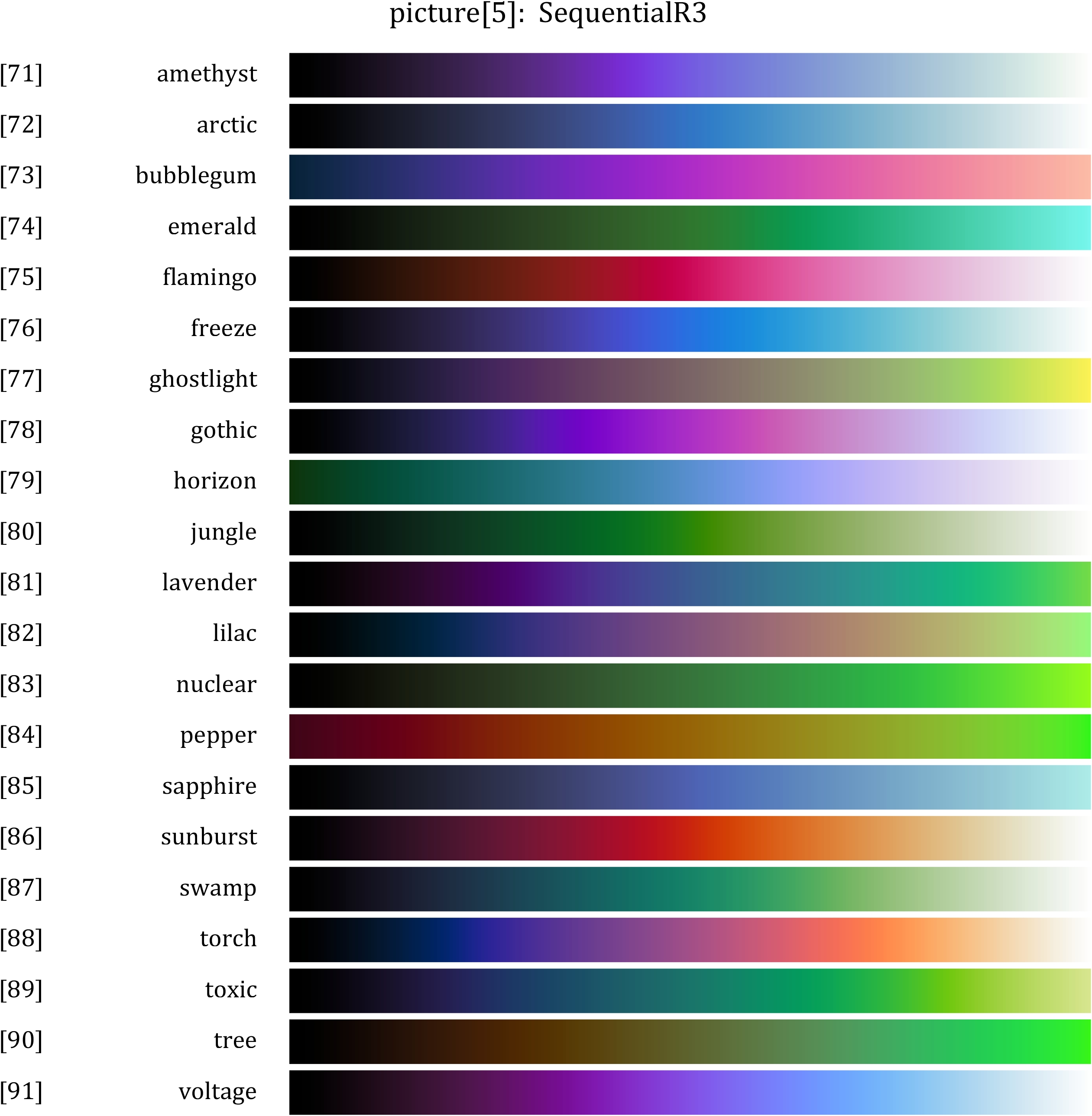

Supplement: Data S1. MATLAB script for ΔFRET map generation [file mmc1.zip › Matlab script/slanCM/slanCM/_5.png]

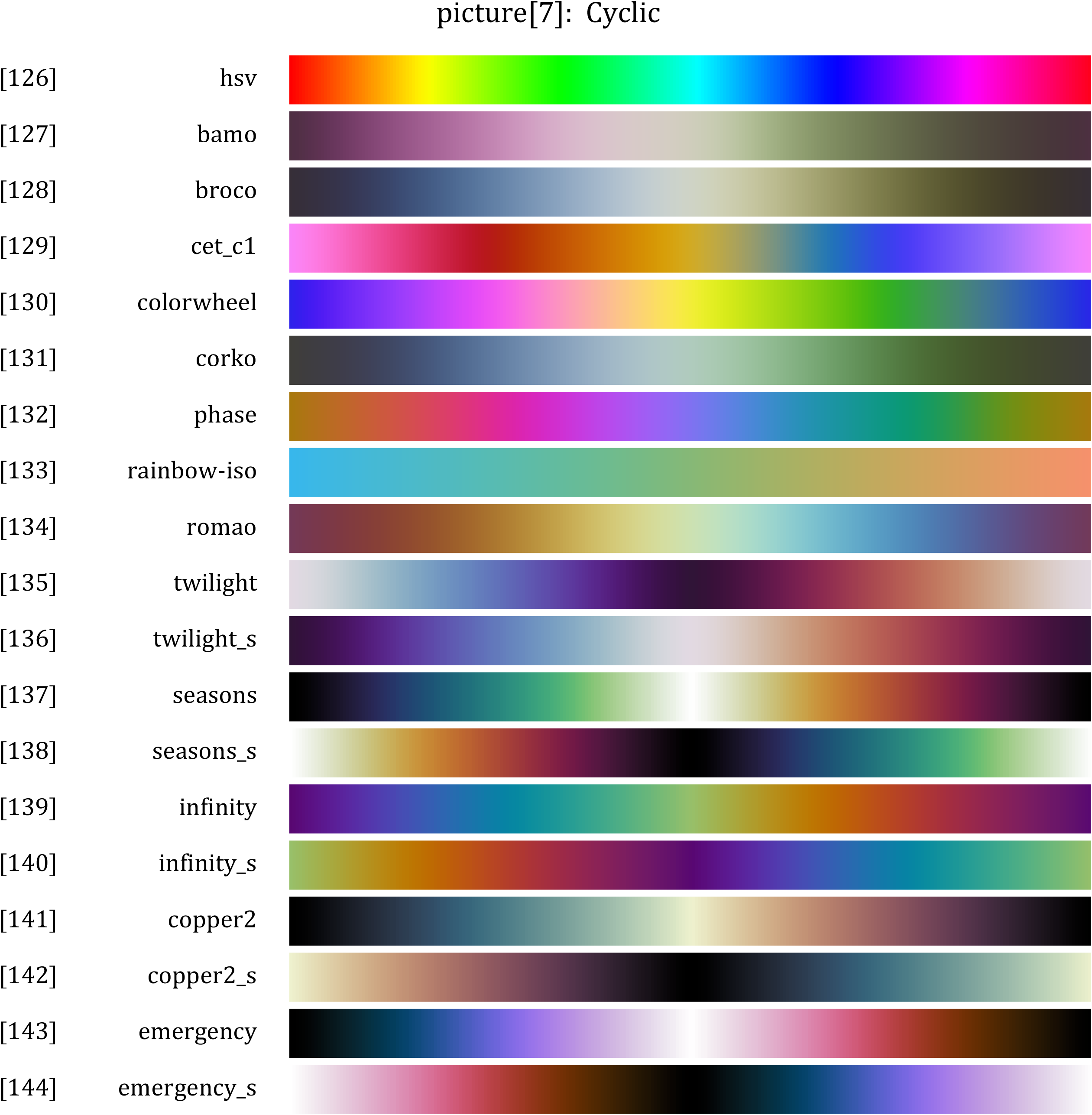

Supplement: Data S1. MATLAB script for ΔFRET map generation [file mmc1.zip › Matlab script/slanCM/slanCM/_7.png]

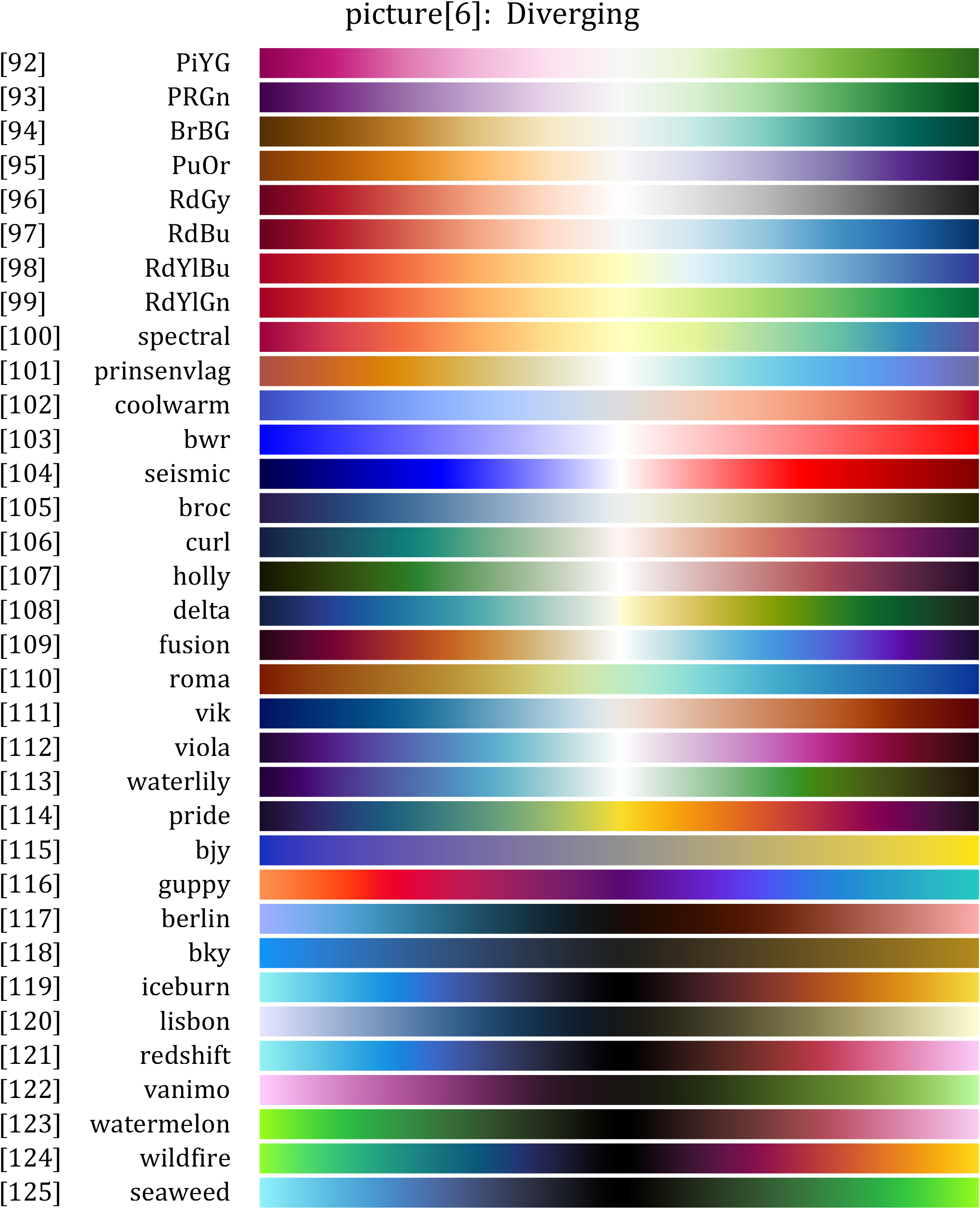

Supplement: Data S1. MATLAB script for ΔFRET map generation [file mmc1.zip › Matlab script/slanCM/slanCM/_6.png]

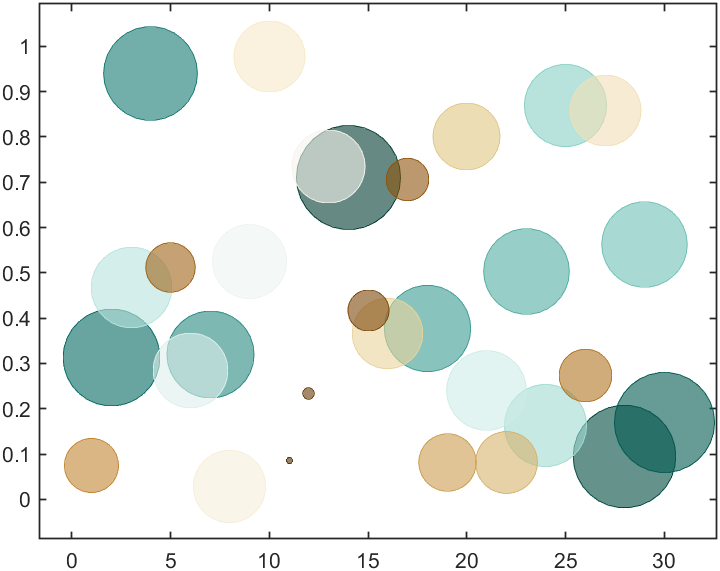

Supplement: Data S1. MATLAB script for ΔFRET map generation [file mmc1.zip › Matlab script/slanCM/slanCM/_demo9_2.png]

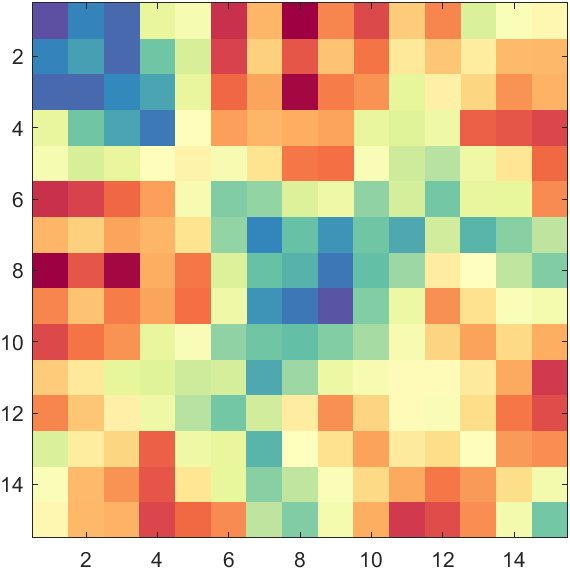

Supplement: Data S1. MATLAB script for ΔFRET map generation [file mmc1.zip › Matlab script/slanCM/slanCM/_demo2.png]

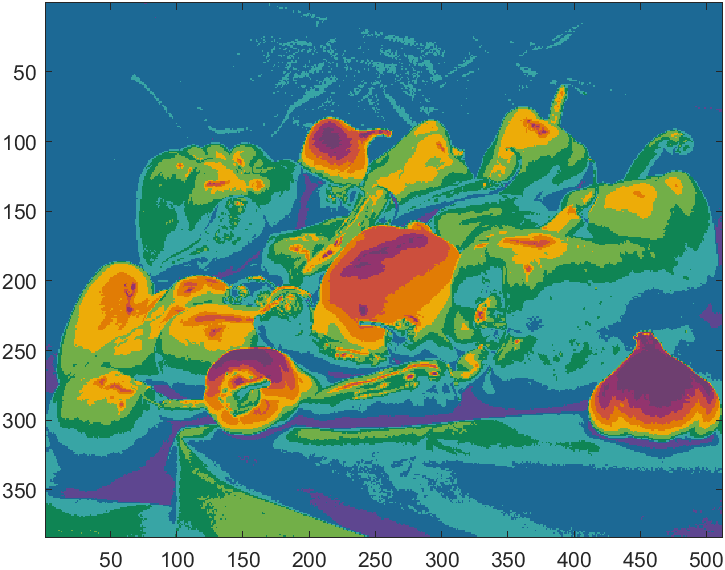

Supplement: Data S1. MATLAB script for ΔFRET map generation [file mmc1.zip › Matlab script/slanCM/slanCM/_demo3.png]

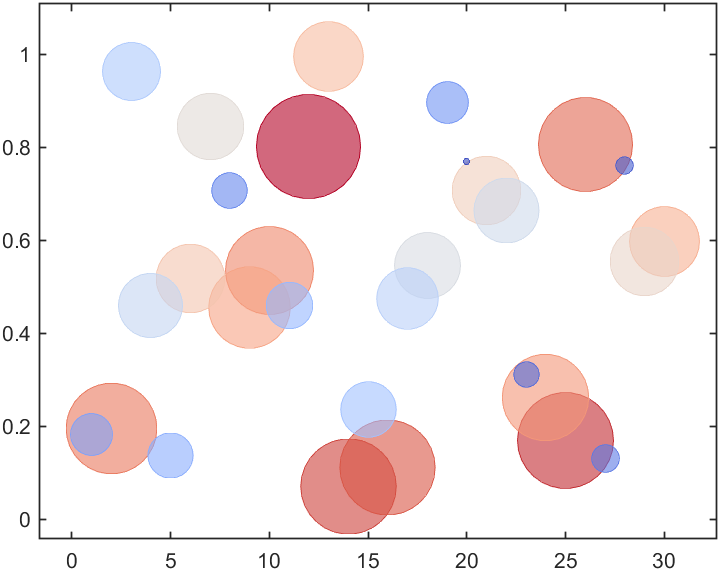

Supplement: Data S1. MATLAB script for ΔFRET map generation [file mmc1.zip › Matlab script/slanCM/slanCM/_demo9_3.png]

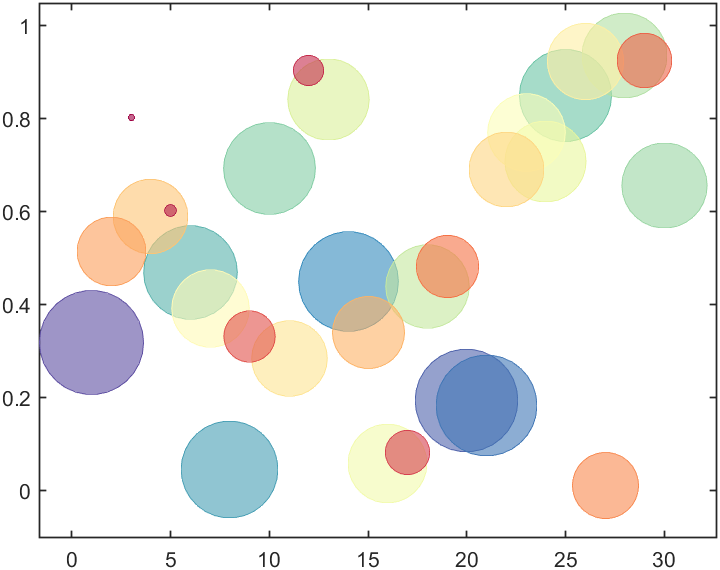

Supplement: Data S1. MATLAB script for ΔFRET map generation [file mmc1.zip › Matlab script/slanCM/slanCM/_demo9_1.png]

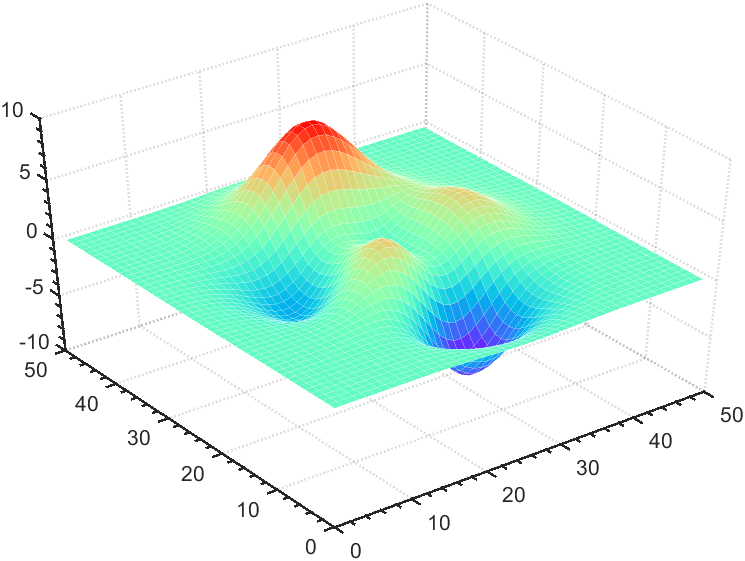

Supplement: Data S1. MATLAB script for ΔFRET map generation [file mmc1.zip › Matlab script/slanCM/slanCM/_demo1.png]

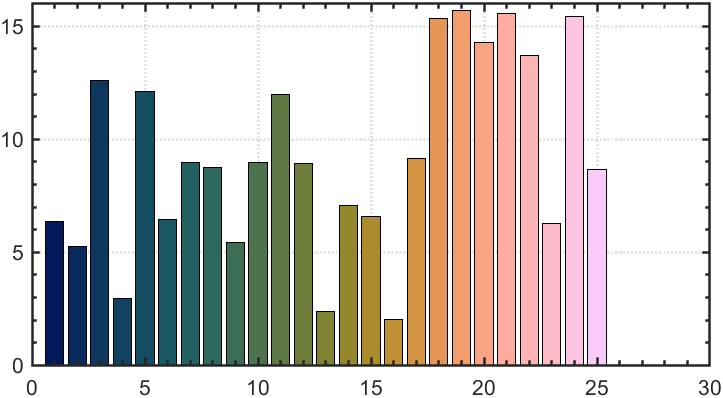

Supplement: Data S1. MATLAB script for ΔFRET map generation [file mmc1.zip › Matlab script/slanCM/slanCM/_demo7.png]

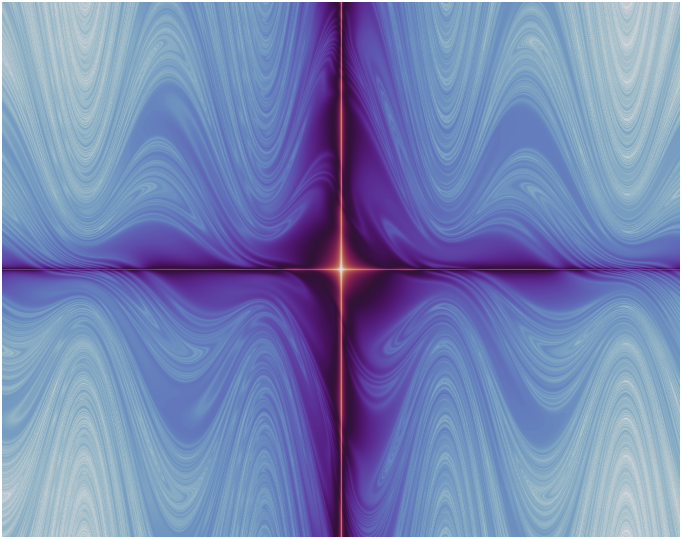

Supplement: Data S1. MATLAB script for ΔFRET map generation [file mmc1.zip › Matlab script/slanCM/slanCM/_demo6.png]
